# Comprehensive longitudinal profiling identifies differential frequency, epitope specificity, and effector function of CD8^+^ T-cells across COVID-19 disease severities

**DOI:** 10.1016/j.ebiom.2026.106310

**Published:** 2026-05-26

**Authors:** Susana Patricia Amaya Hernandez, Kamilla Kjærgaard Munk, Konstantin Danilov, Mohammad Kadivar, Ditte Stampe Hersby, Tripti Tamhane, Simone Majken Stegenborg-Grathwohl, Anders Gorm Pedersen, Anne Ortved Gang, Sine Reker Hadrup, Sunil Kumar Saini

**Affiliations:** a Department of Health Technology, Section of Experimental and Translational Immunology, Technical University of Denmark, Kongens Lyngby, 2800, Denmark; b Department of Hematology, Copenhagen University Hospital, Rigshospitalet, Copenhagen, 2100, Denmark; c Department of Health Technology, Section for Bioinformatics, Technical University of Denmark, Kongens Lyngby, 2800, Denmark; d Department of Clinical Medicine, University of Copenhagen, Copenhagen, 2200, Denmark

**Keywords:** COVID-19, SARS-CoV-2, CD8^+^ T-cells, T-cell epitopes, Viral infections, COVID-19 vaccination, T-cell memory

## Abstract

**Background:**

CD8^+^ T-cells are crucial for controlling and resolving SARS-CoV-2 infection, yet their epitope specificity and relationship to COVID-19 disease severity remain incompletely understood.

**Methods:**

We performed comprehensive longitudinal profiling of antigen-specific CD8^+^ T-cell populations using DNA-barcoded peptide–HLA multimers, analysing 553 SARS-CoV-2 epitopes across globally prevalent HLA alleles in patients with mild and severe COVID-19. Functional and phenotypic characterisation was performed using multidimensional single-cell analysis and detailed cytokine profiling. The impact of post-infection COVID-19 vaccination on T-cell memory was also assessed.

**Findings:**

Severe and mild COVID-19 were associated with robust yet distinct patterns of CD8^+^ T-cell activation. In the acute phase, severe disease was characterised by a broader T-cell repertoire (139 unique epitopes) with a median frequency of 1.4% (IQR 0.2–5.0) and a high-frequency of immunodominant epitope-specific T-cells that exhibited reduced cytotoxic profile. In contrast, patients with mild COVID-19 mounted responses against a more limited set of epitopes (98 unique epitopes), partially overlapping with those observed in severe disease, with a median T-cell frequency of 0.7% (IQR 0–1.9) and displayed a stronger cytotoxic phenotype and functional state. Over time, the memory T-cell compartment contracted to a restricted subset of immunodominant epitopes in the two patient groups and COVID-19 vaccination further enhanced frequencies of spike-specific T-cells independent of prior disease severity.

**Interpretation:**

These findings delineate the epitope-specific frequency, function, and persistence of antigen-specific T-cell populations during SARS-CoV-2 infection, highlighting how differential activation, rather than magnitude alone, shapes immune outcomes across disease severities and other viral infections.

**Funding:**

This work was supported by the Independent Research Fund Denmark (DFF–Sapere Aude, 2066-00044B), the EU Horizon Europe REACT project (101057129), the European Research Council (ERC) Starting Grant MIMIC (101045517), and the Danish National Research Foundation (DNRF170).

**Research in context**

**Evidence before this study**

CD8^+^ T-cells are essential for viral control and protection from severe COVID-19, but functional impairment of these cells has been reported in severe disease. Previous analyses have mainly focused on limited epitope sets or peptide pools, providing incomplete insight into antigen-specific dynamics and memory formation. Consequently, the characteristics of epitope-specific CD8^+^ T-cell populations on disease severity and their persistence after infection or vaccination remain incompletely defined.

**Added value of this study**

This study provides a comprehensive longitudinal map of SARS-CoV-2–specific CD8^+^ T-cell populations across a broad epitope and HLA landscape. By resolving hundreds of epitope-specific populations over time, we identify distinct CD8^+^ T-cell repertoires in the acute phase of infection in patients with mild and severe COVID-19, with patients with severe COVID-19 showing significantly higher proportion of T-cells restricted to immunodominant epitopes. In a selected set of samples, integrating epitope specificity with single-cell transcriptomic, phenotype, and TCR analyses reveal functional differences in T-cells. In severe COVID-19 epitope-specific CD8^+^ T-cells showed reduced activation, whereas mild disease is marked by metabolically active and more cytotoxic T-cells. Together, these findings demonstrate epitope breadth and T-cell quality of antiviral immunity in COVID-19.

**Implications of all the available evidence**

This work defines the relationship between CD8^+^ T-cell specificity, frequency, and function in relation to COVID-19 severity and memory formation. Thus, highlighting quantitative and qualitative differences of T-cell immunity in SARS-CoV-2 infection. By resolving epitope-specific T-cell populations over time, this study provides an immunological framework for understanding disease outcomes and for guiding vaccine design to elicit broad, functional, and protective CD8^+^ T-cell immunity in viral infections.

## Introduction

COVID-19, the disease caused by SARS-CoV-2, presents a broad spectrum of clinical manifestations, ranging from asymptomatic or mild flu-like symptoms, with patients recovering without hospitalisation, to severe pneumonia, acute respiratory distress syndrome (ARDS), and even multi-organ failure, often requiring intensive care and mechanical ventilation.^1^, ^2^, ^3^ The clinical outcomes of COVID-19 are influenced by a combination of risk factors, including age, sex, underlying comorbidities (such as hypertension, diabetes, chronic pulmonary, kidney, or liver disease, immunodeficiencies, cancer, cardiovascular disease, and obesity), and genetic predispositions, which can affect both the viral pathogenesis and the host immune response.^1^^,^^4^ The variable nature of these clinical outcomes emphasises the critical need for a deeper understanding of the immune responses to SARS-CoV-2 infection and the associated disease outcome.

The role of CD8^+^ T-cells has been well documented in mild and asymptomatic SARS-CoV-2 infection and protection from severe COVID-19.^5^, ^6^, ^7^, ^8^, ^9^, ^10^ This is particularly significant considering the observed decline in antibody responses over time^11^, ^12^, ^13^, ^14^ and lesser protection against evolving SARS-CoV-2 variants.^15^^,^^16^ Conversely, dysfunction of CD8^+^ T-cells has also been reported in severe COVID-19, which includes upregulation of exhaustion markers and impaired cytokine production.^17^^,^^18^ Despite the potential dysfunction, we and others have shown robust T-cell activation and a higher frequency of antigen-specific CD8^+^ T-cell populations in patients with severe COVID-19.^14^^,^^19^^,^^20^ Such differential T-cell populations and disease association require a large-scale analysis resolving antigen-specific CD8^+^ T-cells at the early onset of the infection and the influence of such T-cells in establishing long-term memory. However, due to the novel nature of SARS-CoV-2, the antigen-specific immune response and its impact on both short- and long-term immune response remain insufficiently understood.

We have previously shown that in the acute phase of SARS-CoV-2 infection, antigen-specific CD8^+^ T-cell populations are generated against a substantial fraction of SARS-CoV-2 antigens, with higher frequencies of antigen-specific T-cells observed in hospitalised patients compared to patients with mild disease.^19^ In that study, analysis of >3000 peptides, identified 424 immunogenic peptides in COVID-19 patients and in healthy donors.^19^ Similarly, other large-scale analyses of COVID-19 patients have identified a broad range of immunogenic epitopes, constituting more than 1000 CD8^+^ T-cell epitopes in the Immune Epitope Database (IEDB) data repository, covering also variants of SARS-CoV2.^21^ The kinetics of CD8^+^ T-cells in longitudinal analysis ranging from a few weeks to several months has been reported by a few studies.^22^, ^23^, ^24^, ^25^ However, most of the longitudinal analysis followed a few antigen-specific T-cells or performed analysis based on peptide pools, thus providing limited epitope-specific resolution.

In this study, we utilised DNA-barcoded peptide-HLA (pHLA) multimers for a comprehensive longitudinal profiling of 553 SARS-CoV-2-derived CD8^+^ T-cell epitopes in patients with mild and severe COVID-19. Our approach allowed the precise identification and tracking of SARS-CoV-2–specific CD8^+^ T-cells at the level of individual epitopes, enabling us to understand the dynamics of the immune response across different disease severities and to examine the functional and phenotypic differences in CD8^+^ T-cells between these patient groups. To further analyse the immune response, we conducted single-cell profiling of CD8^+^ T-cells targeting SARS-CoV-2 epitopes of interest. By integrating single-cell RNA sequencing, T-cell receptor sequencing, and CITE-seq antibodies, we detailed the phenotypes and functions of specific immune cell subsets, investigating their correlation with COVID-19 severity. We identified distinct epitope-specific CD8^+^ T-cell populations associated with COVID-19 disease severity, with severe disease characterised by broader and higher-frequency T-cell populations but reduced cytotoxic functionality compared to mild disease. Furthermore, we showed that long-term memory is established by a limited set of immunodominant epitopes and can be further enhanced and broadened by COVID-19 vaccination. Overall, our study underlines qualitative and quantitative differences in T-cell immunity across COVID-19 disease outcomes.

## Methods

### Study design

This longitudinal study was designed to investigate the CD8^+^ T-cell mediated immunity against SARS-CoV-2 infection and characterise antigen-specific T-cells in severe and mild COVID-19 disease. We analysed serial samples from 73 individuals with confirmed SARS-CoV-2 infection who presented mild-to-moderate and severe disease (Tables S1 and S2). To study T-cell recognition, immunodominance, long-term memory, and the phenotype of epitope-specific CD8^+^ T-cells, our approach integrated DNA-barcoded peptide-HLA (pHLA) multimers together with a 12-colour flow cytometry panel. To further analyse the immune function, a single-cell analysis was conducted to provide detailed insights into the functionality, transcriptomic profiles, and TCR repertoires of the CD8^+^ T-cells activated during infection. Additionally, our study extended to analyse Spike-specific T-cell populations in 17 SARS-CoV-2 infected patients who were later vaccinated with a COVID-19 mRNA vaccine (Table S2). To complement our analysis of cellular immunity, we employed multiplex bead-based assays to profile antibody levels in plasma. This enabled us to correlate humoural responses with cellular immunity offering a comprehensive view of the immune response to SARS-CoV-2 infection.

### Clinical sample collection and ethical approval

The study protocol, including procedures for sample collection, was approved by the Committee on Health Research Ethics in the Capital Region of Denmark (H-20026375). The patient cohort comprised 73 individuals diagnosed with COVID-19 during the first wave of pandemic (April–May 2020), when the ancestral Wuhan-like virus was predominant. Patients were stratified based on disease severity using an operational classification scheme developed specifically for this study, as no standardise criteria were established at the time. Stratification was based on clinical observations and hospital care requirements, dividing patients into four groups: 1 = non-hospitalised (mild symptoms), 2 = hospitalised with mild symptoms, 3 = hospitalised with severe symptoms, 4 = hospitalised requiring intensive care unit (ICU) support. All patients who required hospitalisation (groups 2, 3, and 4) were classified as severe, except for two patients who were hospitalised due to pre-existing conditions and exhibited no COVID-19 symptoms; these two individuals were categorised as mild. The cohort included 36 patients classified as severe (median age = 62.4 years; mean hospitalisation duration = 8 ± 7.17 days) and 37 patients classified as mild (median age = 47.2 years). Participants were included if they were 18 years or older, had a COVID-19 diagnosis confirmed by RT-PCR testing, and provided written informed consent. Detailed demographic and clinical characteristics of the participants, including age, sex, disease symptoms, disease severity, and comorbidities, were obtained from clinical records and are provided in Table S1. Data were stratified by disease severity group.

Blood samples were systematically collected at four time points. The initial collection (TP1) occurred within two weeks (9 ± 7 days) post-diagnosis. Subsequent collections were scheduled at 27 ± 10 days (TP2), 58 ± 10 days (TP3), and 218 ± 30 days (TP4) after diagnosis. Due to logistical constraints, not all patients were able to provide samples at every designated time point; therefore, some longitudinal data points are missing from the analysis. Between TP3 and TP4, 17 patients received one or two doses of a COVID-19 mRNA vaccine (Pfizer-BioNTech BNT162b2 or Moderna mRNA-1273) (Fig. S1A, Table S2).

Peripheral blood mononuclear cells (PBMCs) were isolated from the collected blood samples by density gradient centrifugation using Leucosep tubes (Greiner Bio-One 227288) with Lymphoprep media (StemCell Technologies 07861). Following isolation, PBMCs were cryopreserved at −150 °C in FCS (Gibco) + 10% DMSO, while the plasma portion was stored at −80 °C for future analysis. Additionally, PBMC samples underwent genotyping for HLA-A, B, and C loci utilising next-generation sequencing techniques (DKMS Life Science Lab GmbH, Germany) (Table S3).

### Quantification of anti-SARS-CoV-2 IgG antibodies using multiplex immunoassay

The multiplex bead-binding assay Bio-Plex Pro Human IgG SARs-CoV-2 N/RBD/S1/S2 4-Plex Panel (Bio-Rad, 12014634) was used to quantify anti-SARS-CoV-2 IgG antibodies specific for Nucleocapsid (N), Spike receptor-binding domain (RBD), Spike 1 (S1) and Spike 2 (S2) antigens. Plasma samples were centrifuged at 1000 × *g* for 10 min before being diluted at a 1:100 ratio using the sample diluent provided by the kit and processed as per manufacturer's instructions for analysis. The assay included both a pre-mixed positive control, comprising human IgG antibodies against the four targeted SARS-CoV-2 antigens, and a negative control to validate the assay's specificity and sensitivity. The sample diluent also served as a blank to measure background non-specific binding levels. All samples and controls were analysed in single measurements. Median fluorescence intensities (MFI) were captured using the Bio-Plex MAGPIX Multiplex Reader (Bio-Rad Laboratories), operated with the xPONENT software version 4.2 (Luminex Corporation). To account for dilution and non-specific background signals, the raw MFI values were adjusted by multiplying by the dilution factor and then subtracting the background MFI.

### SARS-CoV-2 peptide selection

For this study, we selected 553 HLA class I-binding peptides across 9 prevalent HLA alleles, covering 9 proteins from the SARS-CoV-2 isolate Wuhan-Hu-1 (GenBank ID: MN908947.3), previously identified as immunogenic (Tables S4 and S5). Of these, 311 epitopes were identified in our previous study (Saini et al., 2021).^19^

An additional 33 peptides were included based on genome-wide T-cell mapping performed in 19 COVID-19 patients at TP1 using DNA barcoded pHLA multimers (Table S4). In this mapping, 2198 peptides were predicted to bind at least one of seven selected HLA alleles (HLA-A∗01:01, -A∗02:01, -A∗03:01, -A∗24:02, -B∗07:02, -B∗08:01, and -B∗15:01) using NetMHCpan-4.1 with a rank threshold of <1%,^26^ and subsequently screened for CD8^+^ T-cell reactivity (Table S6). The remaining peptides were selected from the Immune Epitope Database (IEDB; www.iedb.org)^21^ based on immunogenic epitopes reported by other studies and restricted to the HLA alleles included in this study with a rank threshold of <1% predicted using NetMHCpan-4.1.^26^ The 553 peptides were used to construct 601 peptide-HLA pairs for experimental assessment (Table S4).

To assess T-cell reactivity in samples from COVID-19 vaccinated individuals, 278 additional Spike peptides (357 pHLA pairs). These peptides were predicted using NetMHCpan-4.1^26^ to bind one or more of the nine prevalent HLA-A and HLA-B molecules, applying a binding rank threshold of <1% (Table S6). This resulted in a comprehensive set of 415 peptides spanning the complete SARS-CoV-2 Spike protein encoded by the Pfizer BioNTech BNT162b2 mRNA vaccine and the Moderna mRNA-1273 vaccine (GenBank ID: QHD43416.1). This expanded set was used to generate 506 Spike peptide-HLA pairs for experimental evaluation at TP4.

Furthermore, 38 peptides from cytomegalovirus (CMV), Epstein–Barr virus (EBV), and influenza (FLU), collectively referred to as CEF peptides, were incorporated to enable comparative analyses with SARS-CoV-2-derived peptide-reactive T-cells (Table S7). The CEF peptide pool represents previously characterised immunodominant CD8^+^ T-cell epitopes.^27^ All peptides were custom-synthesised by Pepscan (Pepscan Presto BV, Lelystad, The Netherlands), dissolved in 10 mM DMSO, and stored at −20 °C until required for experimental procedures.

### HLA class I monomer production

The production of all nine types of HLA proteins followed established protocols.^28^^,^^29^ In summary, recombinant HLA-I heavy chains and human β2-microglobulin (β2m) were produced as inclusion bodies in *Escherichia coli* employing pET series expression vectors. The expressed proteins were refolded with the aid of UV-sensitive peptide ligands specific to each HLA, facilitating the correct assembly of the molecules.^29^ HLA-A∗02:01 and A∗24:02 variants were refolded and purified empty as previously described.^30^ Following the refolding process, the HLA-I molecules were biotinylated utilising the BirA biotin-protein ligase reaction kit (Avidity LLC, Aurora), and then purified using size exclusion chromatography (SEC-HPLC; Waters Corporation, USA). The monomers were stored at −80 °C for future applications.

### Generation of DNA-barcoded multimer libraries

The generation of DNA-barcoded multimer libraries for SARS-CoV-2- and CEF-derived peptides followed the protocol established by Bentzen et al.^31^ Individual pHLA complexes were formed by incubating each peptide with its respective HLA molecule. For HLA-A∗02:01 and A∗24:02, direct peptide loading was employed,^30^ while UV-mediated peptide exchange was used for the remaining HLA types.^29^ The pHLA monomers were then coupled to allophycocyanin (APC) for SARS-CoV-2-derived peptides and phycoerythrin (PE) for CEF-derived peptides using dextran backbones tagged with unique DNA barcodes.

### Antigen-specific T-cell identification

Patient HLA-matching SARS-CoV-2 and CEF pHLA multimer libraries were pooled and incubated with 5 × 10^6^ to 10 × 10^6^ PBMCs for 15 min at 37 °C, as described previously.^31^ This step was followed by a 30-min incubation at 4 °C with a panel of phenotype antibodies and a dead cell marker to assess cell viability (Table S8). After staining, cells were fixed in 1% paraformaldehyde for preservation. Cells were analysed using a FACSAria flow cytometer (AriaFusion, BD Biosciences), where pHLA multimer-binding CD8^+^ T-cells were identified and sorted (Fig. S2A, top).

### Analysis of TCR down-regulation upon antigen stimulation

Ten patient samples (mild, n = 6; severe, n = 4) collected during the early phase post-infection (TP1/TP2) were analysed for activation-induced TCR down-regulation upon SARS-CoV-2 antigen stimulation, as described by Kristensen et al., 2024.^27^ PBMCs from the samples were resuspended in X-Vivo media (Lonza BE02-060Q) supplemented with 5% human serum and stimulated using a SARS-CoV-2 peptide pool including 40 HLA-A∗01:01-, 15 HLA-A∗02:01-, 15 HLA-B∗08:01-, and 15 HLA-B∗35:01-restricted peptides (1 μM of each peptide) (Table S9) or with DMSO (concentration-matched negative control) for 24 h at 37 °C. Following stimulation, cells were washed to remove peptides or DMSO and stained using a pool of DNA barcode-labelled multimers conjugated with PE and APC, prepared as previously described, along with a lineage antibody phenotype panel and a dead cell marker (Table S8). DNA barcode-labelled multimers were generated for the 100 specificities used for stimulation. All double-positive (PE^+^ and APC^+^) pHLA multimer-binding CD8^+^ T-cells were analysed and sorted using a FACSAria flow cytometer (AriaFusion, BD Biosciences). Of the 100 peptides included in the stimulation assay, 17 elicited detectable antigen-specific CD8^+^ T-cell populations in the DNA-barcoded pHLA multimer analysis of the ten selected patient samples and were used in the downstream analysis. The remaining peptides were included to ensure assay robustness.

### DNA barcode sequence analysis

DNA barcodes from sorted multimer positive CD8^+^ T-cell populations, as well as from an aliquot of the multimer pool (to serve as a baseline), were amplified using the Taq PCR Master Mix Kit (Qiagen, 201443) together with sample-specific forward primers to enable accurate sample identification. The amplification products were purified using the QIAquick PCR Purification Kit (Qiagen, 28104) and sequenced at PrimBio (USA) using an Ion Torrent PGM 316, 318, or an Ion S5 530 chip (Life Technologies).

Sequencing data derived from DNA barcodes were analysed using the Barracoda software package (https://github.com/SRHgroup/Barracoda-2.0).^31^ This tool was employed to determine the number of sequencing reads and clonally reduced reads associated with each pHLA-specific DNA barcode. It also calculated the fold changes (FC) in read counts for each sample relative to the average counts from triplicate baseline samples, along with the p-values and false discovery rates (FDRs), as detailed in.^31^ Criteria for defining significant antigen-specific T-cell populations were based on enrichment of DNA barcodes having an FDR <0.1% (equivalent to p < 0.001) and a Log_2_ FC > 2 when compared to baseline values across the entire pHLA library. The frequency of T-cells for each significantly enriched barcode was calculated from its percentage read count relative to the total CD8^+^ multimer^+^ T-cell population. To ensure specificity in the detection of antigen-specific T-cell populations, a non-HLA-matching, non-SARS-CoV-2 infected healthy donor was included as a negative control. Peptides identified in this control sample were excluded from the analysis.

In our analysis of infection-specific CD8^+^ T-cell populations, we excluded Spike-specific populations that demonstrated an increased frequency at TP4 compared to TP3 in patients who were vaccinated between TP3 and TP4. This approach was taken to remove the contribution of vaccine-induced T-cell populations at TP4 from our evaluation.

### Flow cytometry analysis

Flow cytometry data were analysed using FlowJo data analysis software (version 10.9.0; FlowJo LLC). The phenotype analysis of SARS-CoV-2 and CEF pHLA multimer positive CD8^+^ T-cells was conducted according to the gating strategy shown in Fig. S2A. FCS files of samples were concatenated at both the APC-positive and negative-population gates (500 events for each per sample). Concatenated files were visualised using Uniform Manifold Approximation and Projection (UMAP, Version 3.3.3, FlowJo plugin)^32^ and clustered by FlowSOM (FlowJo plugin)^33^ analysis based on the markers CD38, CD39, CD69, CD137, HLA-DR, PD-1, CCR7, CD45RA, and CD27. A heatmap of the FlowSOM metaclusters was automatically generated by the plugin to display the relative expression of each marker within each metacluster. The heatmap was adjusted to show the expression in each cluster relative to the mean fluorescence intensity (MFI) values of each marker, instead of relative to the MFI values of all markers.

### T-cell staining with fluorochrome-labelled pHLA multimers

PBMCs from six COVID-19 patients (mild, n = 3; severe, n = 3), collected during the early phase post-infection (TP1/TP2), were analysed using single- or dual-fluorophore-labelled pHLA multimers. Multimers were prepared as previously described for six selected epitopes: HLA-A∗01:01–restricted TTDPSFLGRY, FTSDYYQLY, VATSRTLSYY, and CTDDNALAYY; HLA-B∗07:02–restricted SPRWYFYYL; and HLA-A∗02:01–restricted YLQPRTFLL. Cells were stained with the fluorophore-labelled pHLA multimers along with a lineage antibody phenotype panel and a dead cell marker (Table S8), and analysed on a FACS Aria flow cytometer (AriaFusion, BD Biosciences). These frequencies were used to validate the corresponding T-cell frequencies estimated by DNA-barcoded pHLA multimer analysis via Spearman's rank correlation. For this comparison, DNA barcoded multimer-derived frequencies represented the summed frequencies of antigen-specific T-cell populations recognising each immunodominant peptide and its corresponding variant peptides. The following peptide variants were included: for TTDPSFLGRY — HTTDPSFLGRY, TTDPSFLGRYM; for FTSDYYQLY — YFTSDYYQLY; for CTDDNALAYY — CTDDNALAYYN, TDDNALAYY; and for VATSRTLSYY — ATSRTLSYY.

### Data processing and statistics

T-cell recognition data, determined by DNA-barcoded pHLA multimer analysis and processed using the Barracoda software, were plotted using R Studio.^34^ The ggplot2 package (version 3.4.4)^35^ was used to generate box, bar, column, scatter, and dot plots for data visualisation, and Venn diagrams were generated using the eulerr package (version 7.0.0).^36^

In dot plots for visualisation of antigen-specific T-cell populations, peptide sequences with no significant enrichment are shown as grey dots, and peptides with negative enrichment are set to a log_2_ fold change equal to zero. Significant antigen-specific T-cell populations were coloured according to plot-specific criteria. The size of each dot is proportional to the estimated frequency (%), calculated from the percentage read count of the associated barcode relative to the total CD8^+^ multimer^+^ T-cell population.

Box plots were generated for data quantification and visualisation, and statistical analyses were performed using the package rstatix (version 0.7.2).^37^ The Mann–Whitney test was used for unpaired comparisons, and p-values were adjusted using the Bonferroni method when comparisons across multiple time points were performed. Spearman's rank correlation coefficient (ρ) and the corresponding P-value were used to assess correlations. Fisher's exact test with Hochberg-Benjamini multiple testing correction (significance level p < 0.05) was conducted using the package stats version 4.3.2.^31^ Multimer^+^ CD8^+^ T-cells (SARS-CoV-2- or CEF-specific) are reported as frequencies (% of total CD8^+^ T-cells). Values throughout the study are reported as mean ± standard deviation (SD) or median (interquartile range (IQR)), as appropriate.

### Bayesian mixed-effects binomial logistic regression

#### Model overview

The aim of this statistical analysis was to test the hypothesis that T-cell reactivity in severe COVID-19 involves a different set of epitopes and/or HLA alleles than in mild disease, while accounting for (i) which peptide-HLA specificities were testable in each patient and (ii) patient-to-patient heterogeneity in overall responsiveness. The data were analysed using a hierarchical (mixed-effects) binomial logistic regression model. Each observation *n* corresponds to one testable patient x peptide-HLA multimer measurement (i.e., a peptide presented by an HLA allele carried by that patient). For each patient sample, the number of CD8^+^ T-cells positive for the corresponding peptide-HLA multimer, *y*_*n*_, and the total number of CD8^+^ T-cells measured, *n*_*n*_ were recorded. The multimer^+^ fraction among CD8^+^ T-cells cells was modelled as:

$$y_{n}∼\text{Binomial}\left(n_{n},p_{n}\right),\text{logit}\left(p_{n}\right)=\eta_{n}$$

where *p*_*n*_ is the expected pHLA^+^ (multimer^+^) fraction of CD8^+^ T-cells for that patient–peptide–HLA observation. Observations were only available for peptide–HLA pairs present in the multimer panel and for which the patient carried the relevant HLA allele; other combinations are structurally missing rather than true zeros.

In the linear predictor, each observation *n* is linked to a patient/sample *s*[*n*], a peptide *p*[*n*], and an HLA allele *a*[*n*]. A peptide-HLA pair index *j*[*n*] was also defined for the specific (*p*[*n*],*a*[*n*]) combination. Disease severity was defined at the sample level, with *sev*_*s*_ ∈ {0,1} for mild and severe disease, respectively.

The linear predictor was written as a baseline component plus a severity-dependent component:

$$\eta_{n}=\eta_{n}^{\left(0\right)}+\;sev_{s\left[n\right]}\;\eta_{n}^{\left(\text{sev}\right)}$$

with the baseline component defined as:

$$\eta_{n}^{\left(0\right)}=\;b_{0}+\;b_{\text{sex}}\;\text{se}x_{s}\;+\;B_{\text{age}}{\left(s\right)}^{⊤}b_{\text{age}}\;+\;u_{s}\;+\;\alpha_{p\left[n\right]}\;+\;\gamma_{a\left[n\right]}\;+\;\delta_{j\left[n\right]}$$

and the severity-dependent shift defined as:

$$\eta_{n}^{\left(\text{sev}\right)}=\;b_{\text{sev}}\;+\;\alpha_{p\left[n\right]}^{\left(\text{sev}\right)}\;+\;\gamma_{a\left[n\right]}^{\left(\text{sev}\right)}$$

Here *b*_0_ is a global intercept (baseline log-odds of a pHLA^+^ event in the reference group); *b*_sex_ is the regression coefficient for sex (with sex_s_ = 1 for male and 0 for female); *B*_age_(*s*)^⊤^*b*_age_ represents a flexible age-effect modelled using a spline basis; and *u*_*s*_ is a sample/patient-specific random intercept that captures overall differences in responsiveness across patients (biological and/or technical). The terms α_*p*_ and γ_*a*_ are peptide- and HLA-allele-specific effects in mild patients, while $\alpha_{p}^{\left(\text{sev}\right)}$ and $\gamma_{a}^{\left(\text{sev}\right)}$ allow these peptide and allele effects to shift in severe disease. Finally, δ_*j*_ is a peptide-HLA pair-specific random effect that captures residual interaction not explained by the additive peptide and allele components. In this model, the pair effect δ_*j*_ is not severity-dependent; instead, severity-associated differences are represented through a global severity shift *b*_sev_ and peptide- and allele-specific severity shifts.

Overall, this structure separates (i) baseline differences between patients, (ii) peptide-level and allele-level tendencies that generalise across the cohort, and (iii) residual pair-specific deviations, while allowing peptide and allele effects to change with severity. This enables testing whether severe disease is associated with a different pattern of epitope-specific CD8^+^ T-cell responses than mild disease, while accounting for which peptide-HLA specificities were testable in each patient and for substantial patient-to-patient heterogeneity.

As a sensitivity analysis, an analogous Bernoulli (binary) version of the model was fitted using response presence or absence per observation. This produced qualitatively similar severity-associated patterns but with wider uncertainty, consistent with reduced information relative to the count-based model.

#### Bayesian inference and priors

The model was fitted in a Bayesian framework. Fixed-effect coefficients were assigned weakly informative Normal priors centred at 0 (intercept $b_{0}∼N\left(0,1.5\right)$; remaining fixed effects ∼N(0,0.7)). Random effects (patient, peptide, allele, and peptide-HLA pair) were modelled as mean-zero Normal deviations with group-specific standard deviations, estimated with weakly informative half-Normal priors. This hierarchical structure induces partial pooling, stabilising estimates for sparsely observed peptides, alleles, and pairs and shrinking unsupported effects toward zero.

Posterior sampling was performed using Hamiltonian Monte Carlo (No-U-Turn Sampler) as implemented in Stan via CmdStanR.^38^ Convergence and sampling quality were assessed using $\hat{R}$, effective sample sizes, and standard Stan diagnostics (divergences, treedepth, and E-BFMI).

#### Summarising severity-associated changes using average predictive comparison

To present severity effects on an interpretable probability scale, the fitted model was summarised using average predictive comparisons (AvgPC).^39^ For each posterior draw and each observation n, two predicted response probabilities were predicted:

• *p*_*n*,0_: predicted probability if severity were set to mild (*sev* = 0),
• *p*_*n*,1_: predicted probability if severity were set to severe (*sev* = 1),

while holding all other predictors (patient identity, age, sex, peptide, allele, and peptide-HLA pair) at their observed values.

The severity-associated change was then computed as Δ*p*_*n*_ = *p*_*n*,1_−*p*_*n*,0_ and averaged Δ*p*_*n*_ over observations belonging to a given peptide, allele, or peptide-HLA pair to obtain peptide-, allele-, or pair-specific AvgPCs. Posterior medians, credible intervals, and posterior probabilities (e.g. *Pr*(Δ*p* > 0)) are reported.

Unless otherwise stated, AvgPCs were computed using observation-weighted averaging, such that each testable observation contributes equally.

### T-cell staining and sorting for single-cell analysis

Six COVID-19 patients (severe, n = 3; mild, n = 3; Table S10), with detectable antigen-specific T-cell populations identified through DNA barcode-labelled pHLA multimers were selected for single-cell analysis. For each patient a sample collected early after SARS-CoV-2 infection (TP1 or TP2) and a sample collected at a late time point (TP4) were included for analysis. PBMCs from the selected samples were cultured in X-vivo media +5% HS and stimulated with 1 μM of SARS-CoV-2 peptides for 24 h at 37 °C, 5% CO_2_. Refer to Table S10 for details on peptides used for each sample. Following peptide stimulation, cells were washed with Cell Staining Buffer (PBS + 0.5% BSA) and resuspended in 20 μL of the buffer. Five μL of Human TruStain FcX Fc Blocking reagent was added to the cells, and incubated 10 min at 4 °C. The TotalSeq-C Human Universal Cocktail (BioLegend 399905) phenotype panel was reconstituted with 27.5 μl of cell staining buffer. Subsequently, 12 μl of the reconstituted cocktail were added to each sample and incubated for 15 min at 4 °C. Then, 0.5 μl of hashing antibodies (BioLegend, TotalSeq-C anti-human Hashtag 1–16 Antibodies, Table S10), an antibody solution mix and a dead cell marker (Table S8) were added to each sample and incubated for 30 min at 4 °C. Cells were washed three times in cell staining buffer and maintained on ice until acquisition. Activated CD8^+^ T-cells were sorted based on the activation markers CD69 and CD137 using a FACS Melody (BD) (gating strategy provided in Fig. S7). Approximately 20,000 cells from all samples were collected into a single tube containing 100 μL of Cell Staining Buffer, centrifuged at 390 g for 10 min at 4 °C, and the supernatant discarded.

### Construction of single-cell libraries

The preparation of Gel Beads-in-emulsion (GEMs) and downstream processing of DNA barcodes and mRNA was performed utilising the 10x Genomics 5′ v2 chemistry according to the manufacturer's protocol (Chromium Next GEM Single Cell 5′ Reagent Kits v2 (Dual Index), with the Feature Barcode technology for Cell Surface Protein & Immune Receptor Mapping) (10x Genomics, USA). Sorted cells were loaded into a single lane of a Chromium Next Generation Chip and run in a Chromium Controller (10x Genomics, USA) to generate individual GEMs, followed by synthesis of cDNA from poly-adenylated mRNA, and DNA from cell surface protein Feature Barcode derived from antibodies. After 16 cycles of targeted amplification, the products were separated according to size using SPRIselect beads (Beckman Coulter, B23318) and processed separately for the construction of TCR (VDJ), 5ʹ Gene Expression (GEX), and Barcode (BC) libraries. The libraries were quantified with the Qubit dsDNA HS Assay Kit (Invitrogen Q32851) and combined at a ratio of 1 BC: 5 GEX: 1.5 TCR. Libraries were sequenced at Novogene Company Limited (UK) on a NovaSeq system (Ilumina) running a 150 paired-end program.

### Single-cell RNA and ADT analysis

Raw FASTQ files were aligned to the human reference genome GRCh38 v1.2.0 and V(D)J reference v.5.0 with Cell Ranger v.7.1.0^40^ using command cellranger multi with default parameters. Reference for the feature layer was constructed according to the barcodes that have been used in the experiment. The resulting matrices were loaded as a Seurat object (Seurat v5.0.2)^41^ and analysed. Demultiplexing was performed using HTODemux with standard parameters after Centered Log-Ratio (CLR) normalisation for hashing antibodies with more than 10.000 read counts. Next, singlets were filtered to exclude cells with more than 2000 or less than 200 expressed genes with ≥5% detected mitochondrial genes. Only protein coding (biomaRt v2.58.2)^42^ and mitochondrial genes were considered for gene expression assay. We retained all cells that passed transcriptomic quality control for downstream analysis, irrespective of TCR capture status. Subsequently, we applied CLR normalisation for the antibody derived tag (ADT) assay and the LogNormalize method for gene expression assay.

After scaling, 2000 most variable genes were selected for the PCA, and 18 principal components were used for clustering and UMAP visualisation. We noticed that the first principal component is correlated with nFeature RNA metrics and affects further steps and clustering, thus, we concluded that resulting clusters are more likely related to technical variation in our data rather than biological one and we focused on a comparison between conditions (patients with mild vs severe COVID-19 and early vs late time points) instead of cluster-based analysis. Also, a small cluster of cells was found with high expression of heat shock proteins and excluded for further analysis (cluster 5, Fig. S9D).

Gene expression markers for the resulting clusters were calculated using FindAllMarkers with minimal percentage = 0.25 and logFC threshold = 0.25. Differentially expressed genes between conditions were calculated on a cell basis (FindMarkers, minimal percentage = 0, logFC threshold = 0) or using pseudobulk approach with AggregateExpression and DESeq2 (v1.42.1).^43^ Gene set enrichment analysis^44^ was applied to ranked statistics after differential expression on cell level (−log10(p_val)∗sign(avg_log_2_FC)) using fgsea package^45^ (unreviewed preprint) and gene sets from MSigBD (hallmark gene sets)^44^ and selected publications (Li et al.,^46^ Gangaev et al.,^47^ Cai et al.^48^). We used ComplexHeatmap^49^ and EnhancedVolcano^50^ packages for the visualisation of differential expression analysis results.

### TCR profiling data analysis

Package scRepertoire^51^ was used to analysed the filtered contig annotations. We called clones “strict” - VDJC gene and CDR3 nucleotide and set the filterMulti as TRUE, allowing us to select the top 2 represented chains for cells with multiple chains.

### Single-cell statistics

Mann–Whitney test was used to compare two independent groups. Analyses were performed in R Studio version 4.3.3.^34^

### Functional characterisation of T-cells using the Olink platform

Eighteen patient samples (mild, n = 9 (mean age 50.7 ± 13.8 years; 4 females, 5 males); severe, n = 9 (mean age 59.8 ± 15.8 years; 4 females, 5 males)) collected at early phase post-infection (TP1/TP2), along with two additional samples from patients with severe disease collected at a late phase (TP4; samples AP-03 (male, 55 years) and AP-75 (male, 35 years)), were analysed using proximity extension assay technology (Olink Proteomics AB, Sweden). PBMCs (1 × 10^6^ cells) were cultured in X-vivo media supplemented with 5% HS and incubated for 24 h at 37 °C. After incubation, cells were washed three times with PBS to remove residual HS, resuspended in X-vivo media, and stimulated with either 1 μM of individual SARS-CoV-2 epitopes or DMSO (concentration-matched negative control) for an additional 24 h at 37 °C (Table S11). Following stimulation, cell supernatants were collected for analysis. The DTU Centre for Diagnostics (Denmark) conducted the analysis using the Olink® Target 48 Cytokine panel (Olink Proteomics AB, Sweden), which enables simultaneous quantification of 45 inflammatory protein biomarkers. Quality control was ensured by running the Olink® Target 48 Sample Control in triplicate. Each sample was analysed in a single measurement, and protein concentrations were reported in pg/mL.

For data analysis, protein concentrations from DMSO-stimulated samples were subtracted from those of SARS-CoV-2 peptide-stimulated samples to determine the specific response to viral epitopes. Measurements exceeding the upper limit of quantification (ULOQ) were set to the ULOQ value for the respective protein, while those with NaN readings were adjusted to 0 pg/mL. Normalisation was performed based on the proportion of peptide-specific T-cells in each sample to determine the expected protein concentration per 100 cells (Table S14).

Data visualisation was performed using the ggplot2 package (version 3.4.4)^35^ in R Studio. For dot plot visualisations, numerical data were row-scaled to a range of 0–1 to facilitate comparison and interpretation. Scaling was performed using the rescale function from the scales package (version 1.3.0) in R Studio. Differences between severe and mild COVID-19, as well as early and late conditions, were analysed by calculating fold changes for each analyte. For fold-change analyses Fig. S16, normalised protein concentrations were log_2_-transformed after addition of a pseudocount (0.001) to account for zero values. Log_2_ fold changes were calculated as the difference between mean log_2_-transformed values of the respective groups, corresponding to the log_2_ ratio of geometric means. Ninety-five percent confidence intervals (95% CI) for the log_2_ fold changes were calculated based on the standard error of the difference between group means on the log scale. Statistical significance was assessed using the Mann–Whitney test.

### Role of funders

The funders of the study had no role in experimental design, data collection, data analysis, data interpretation, or writing of the study.

## Results

### COVID-19 patient cohort and selection of T-cell epitopes

For the longitudinal analysis of CD8^+^ T-cells in COVID-19 disease severity and their long-term implications, we enrolled 73 patients infected during the first wave of the pandemic and followed them for approximately 220 days. Patients were stratified based on clinical observations and hospital care requirements into four groups: (1) non-hospitalised with mild symptoms, (2) hospitalised with mild symptoms, (3) hospitalised with severe symptoms, and (4) hospitalised requiring intensive care unit (ICU) support. For analysis, all hospitalised patients (groups 2–4) were classified as severe, except for two individuals who were admitted due to pre-existing conditions but displayed no COVID-19 symptoms; these were categorised as mild. In total, the cohort consisted of 36 patients classified as severe (median age = 62.4 years; mean hospitalisation duration = 8 ± 7.17 days) and 37 patients classified as mild (median age = 47.2 years) (Table S1).

**Fig. 1:**
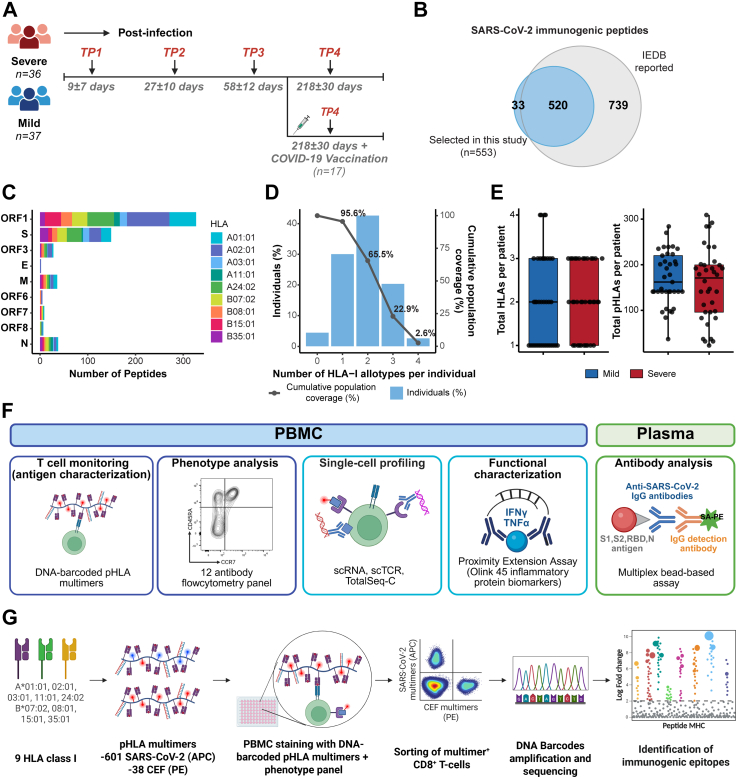
**Study design and selection of SARS-CoV-2 immunogenic peptides for longitudinal analysis.** (**A**) Sample collection timeline for patients with COVID-19 (severe, n = 36; mild, n = 37; TP: time point). Vaccinated patients received 1 or 2 doses of a COVID-19 vaccine (n = 17). (**B**) Venn diagram shows the number of SARS-CoV-2 derived epitopes included in this study (n = 553) out of the total SARS-CoV-2 epitopes reported in IEDB. (**C**) Distribution of the selected SARS-CoV-2 epitopes among the structural proteins (S, M, E, N) and non-structural proteins (ORF1, ORF3, ORF6, ORF7, and ORF8) according to their HLA-restriction. (**D**) Population coverage achieved with the selection of nine common HLA class I allotypes for SARS-CoV-2 T-cell epitope screening and analysis. The percentage of individuals within the world population carrying up to four HLA-I allotypes (*x-*axis) are indicated as blue bars on the left *y-*axis. The cumulative percentage of population coverage is depicted as grey dots on the right *y-*axis. (**E**) Comparison of the total number of HLA (**left**) and pHLA (**right**) included for analysis per patient between the severe and mild groups. Each dot represents one patient. Mann–Whitney test, mild vs severe HLA (p = 0.718), mild vs severe pHLA (p = 0.456). (**F**) Schematic diagram illustrating the different assays used in this study. (**G**) Experimental workflow for analysing T-cell recognition of immunogenic SARS-CoV-2–derived HLA-binding peptides in PBMCs using DNA-barcoded pMHC multimers.

Following PCR-confirmed SARS-CoV-2 infection, during the first wave of the COVID-19 pandemic (April–May 2020), blood samples were collected at four longitudinal time points (TPs): TP1 (9 ± 7 days post-diagnosis, as close as possible to the positive test; severe n = 34 (mean age 58.5 ± 15.1 years (14 females, 20 males)), mild n = 36 (mean age 46.2 ± 12.9 years (19 females, 17 males))), TP2 (27 ± 10 days; severe n = 22 (mean age 58.8 ± 12.3 years (10 females, 12 males)), mild = 32 (mean age 49.0 ± 13.8 years (18 females, 14 males))), TP3 (58 ± 12 days; severe n = 19 (mean age 59.8 ± 13.7 years (11 females, 8 males)), mild n = 29 (mean age 48.3 ± 12.1 years (15 females, 14 males))), and TP4 (218 ± 30 days; severe n = 23 (mean age 59.3 ± 12.2 years (11 females, 12 males)), mild n = 35 (mean age of 46.3 ± 13.0 years (19 females, 16 males))), extending until January 2021. This represented the first documented SARS-CoV-2 infection for all participants, and no additional SARS-CoV-2 infections were reported during the follow-up period between sampling time points. Within this cohort, 17 individuals (severe, n = 10; mild, n = 7) received one or two doses of a COVID-19 mRNA vaccine between TP3 and TP4; no participants received COVID-19 vaccination prior to infection or during the study period outside this interval (Fig. 1A, Fig. S1A, Table S2).

To comprehensively cover the SARS-CoV-2 CD8^+^ T-cell epitope landscape, we selected a total of 553 unique epitopes reported by us and others (Fig. 1B, Table S4). Of these, 311 epitopes were identified in our previous study.^19^ Additionally, 2198 SARS-CoV-2 peptides predicted to bind at least one of seven selected HLA alleles (HLA-A∗01:01, -A∗02:01, -A∗03:01, -A∗24:02, -B∗07:02, -B∗08:01, and -B∗15:01) using NetMHCpan-4.1 with a rank threshold of <1%,^26^ were used for T-cell response analysis using DNA-barcoded pHLA multimers in 19 COVID-19 patients (Table S6). From this analysis, 33 peptides that elicited detectable CD8^+^ T-cell populations in patient samples were selected for the longitudinal analysis (Table S4). The remaining epitopes were selected from the Immune Epitope Database (IEDB), and restricted to the HLA alleles included in this study, using the same rank threshold of <1% (NetMHCpan-4.1).^21^^,^^26^ Altogether, the 553 epitopes represented nine of the most prevalent HLA alleles in Caucasian population (HLA-A∗01:01, -A∗02:01, -A∗03:01, A∗11:01, -A∗24:02, -B∗07:02, -B∗08:01, -B∗15:01, and -B∗35:01) and spanned nine SARS-CoV-2 proteins (Fig. 1C, Tables S4 and S5). This epitope set provided global population coverage, with at least one HLA allotype represented in 95.6% of the global population (Fig. 1D). All study participants were HLA typed, and epitope selection and pHLA multimer analyses were performed based on each donor's HLA class I genotype (Table S3). On average, HLA coverage was 2.08 alleles per individual in patients with severe COVID-19 and 2.05 alleles in patients with mild COVID-19 (Fig. 1E, Table S3).

We used DNA-barcoded pHLA multimers to longitudinally profile CD8^+^ T-cell reactivity.^31^ Because some epitopes were restricted by more than one HLA allele, the 553 peptides corresponded to 601 peptide–HLA combinations for experimental assessment (Table S4). DNA-barcoded multimers were prepared by loading HLA-specific peptides onto HLA molecules, followed by multimerization on PE (phycoerythrin)– or APC (allophycocyanin)– labelled dextran backbones tagged with unique DNA barcodes.^31^ Peripheral blood mononuclear cells (PBMCs) from each patient were incubated with HLA-matching pHLA multimers (Table S3) and stained with a phenotype antibody panel (Table S8) to identify multimer-reactive CD8^+^ T-cells (Fig. 1F and G). On average, 159 (±79.8) and 172 (±59.5) pHLA multimers were analysed in patients with severe and mild disease, respectively (Fig. 1E). Additionally, for comparisons between SARS-CoV-2 and other well-characterised viral antigens, 38 epitopes derived from cytomegalovirus (CMV), Epstein–Barr virus (EBV), and influenza (FLU), collectively referred to as CEF, were also included in the analysis (Table S7).

### Differential T-cell activation in mild and severe COVID-19

**Fig. 2:**
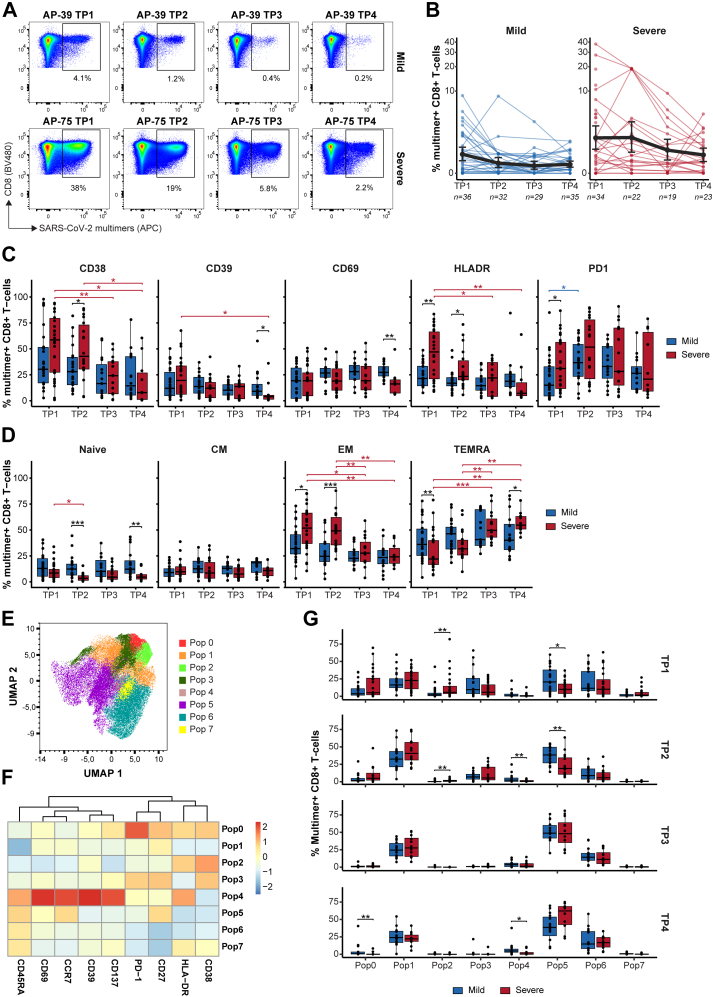
**Longitudinal memory and activation profiles of SARS-CoV-2-specific CD8^+^ T-cells in patients with mild and severe COVID-19.** SARS-CoV-2–specific CD8^+^ T-cells were identified using DNA-barcoded pHLA multimer technology and analysed by flow cytometry across four longitudinal time points. (**A**) Representative flow cytometry plots of SARS-CoV-2-specific multimer^+^ CD8^+^ T-cells from a patient with mild (**top**) and severe (**bottom**) COVID-19 across four time points. Gated populations were sorted for DNA barcode analysis to determine epitope specificity. (**B**) Kinetics of the frequency (% pHLA multimer^+^ CD8^+^ T-cells) in patients with mild (**left**) and severe (**right**) COVID-19 across four time points (TP1–TP4). Each line represents longitudinal measurements from an individual patient, and the bold black line indicates the mean frequency for each group. Unconnected points indicate samples from individuals with missing time points in the longitudinal follow-up. (**C**) Box plots indicating the percentage of pMHC multimer^+^ CD8^+^ T-cells expressing CD38, CD39, CD69, HLA-DR, and PD-1. (**D**) Memory phenotype (Naive, CM, EM and TEMRA) of pHLA multimer^+^ CD8^+^ T-cells based on the cell surface expression of CD45RA and CCR7. (**E**) UMAP visualisation of SARS-CoV-2 pHLA multimer^+^ CD8^+^ T-cells coloured by FlowSOM-identified populations. (**F**) Heatmap showing mean fluorescence intensity (MFI) of each marker across FlowSOM-identified populations. (**G**) Box plots comparing the distribution of pMHC multimer positive CD8^+^ T-cells between patients with mild and severe COVID-19 for each FlowSOM population across time points. (B, C, D, G) Mann–Whitney test was used for comparison between disease severity, with the Bonferroni correction applied for comparisons between time points, ∗∗∗∗ (p < 0.0001), ∗∗∗ (p < 0.001), ∗∗ (p < 0.01) and ∗ (p ≤ 0.05).

To assess CD8^+^ T-cell activation in relation to disease severity, antibody responses, and initial viral load, we first compared the frequency of total pHLA multimer–reactive CD8^+^ T-cells across all time points. In acute infection, the overall frequency of CD8^+^ T-cells binding to SARS-CoV-2 pHLA multimers was higher in patients with severe COVID-19, reaching up to 38% of the total CD8^+^ T-cells in individual patients, compared with a maximum of 9% in patients with mild COVID-19 (Fig. 2A and B). The SARS-CoV-2-specific T-cell frequencies declined sharply after the acute phase (from TP1 to TP2) in patients with mild disease but not in those with severe disease. The initial high T-cell frequencies in the severe disease group remained higher than the mild disease group at all time points (Fig. 2B). Similar to CD8^+^ T-cells, and in line with published data,^23^^,^^52^^,^^53^ patients with severe COVID-19 also exhibited higher antibody levels across all time points (Fig. S1C; RBD-specific antibodies: TP2 p = 0.00003; TP3 p = 0.003; TP4 p = 0.0065). Furthermore, the frequency of SARS-CoV-2-specific T-cells did not correlate with viral load (at the time of positive PCR test), as patients with severe disease showed significantly lower viral load compared to patients with mild disease (Fig. S1B).

Next, we analysed the phenotype kinetics of multimer-positive CD8^+^ T-cells based on the expression of cell surface markers (Fig. 1F, Fig. S2A). Compared to CEF-specific T-cells, SARS-CoV-2–specific CD8^+^ T-cells exhibited significantly higher expression of activation markers in both mild and severe disease patients with significant increases in CD38, CD39, and HLA-DR in mild disease and CD38, CD39, HLA-DR, and PD-1 in severe disease at TP1, and in CD38, CD39, CD69, and HLA-DR in mild disease at TP4 (all p < 0.001) (Fig. S2B). No differences were observed in the frequency or activation of CEF-specific T-cells between the two groups, except for CD38 expression at TP1 (p = 0.0104) (Fig. S2C and D, Table S12). Notably, SARS-CoV-2-specific T-cells of patients with severe COVID-19 showed strong expression of CD38, HLA-DR, and PD-1 during the acute phase, which gradually decline towards TP4 (TP1 vs TP4: CD38 p = 0.013, HLA-DR p = 0.002). During this phase, expression of these markers was significantly higher in patients with severe disease compared to mild disease (TP1: HLA-DR p = 0.0013, PD-1 p = 0.0256; TP2: HLA-DR p = 0.0311, CD38 = 0.0374). In contrast, the expression of these activation markers was consistently lower in mild cases across time points. CD39 and CD69 levels were similar during the early phase; however, significantly higher expression of these markers was observed in patients with mild disease at TP4 (CD39 p = 0.0309; CD69 p = 0.0022) (Fig. 2C). Across all time points, the majority of these T-cells were either effector memory (EM; CD45RA^low^, CCR7^low^) or terminally differentiated effector memory cells re-expressing CD45RA (TEMRA; CD45RA^hi^, CCR7^low^), with low frequency of circulating naïve and central memory (CM) cells (Fig. 2D). We also observed relatively high proportion of naïve T-cells (CD45RA^hi^, CCR7^hi^) most likely represent antigen-experienced naïve-like memory or stem-cell memory T-cells.^54^^,^^55^ Noticeably, at the acute phase of infection (TP1), T-cells of patients with mild disease displayed a significant enrichment of the TEMRA subset compared to patients with severe disease (p = 0.0086). In contrast, TEMRA acquisition in patients with severe disease was slower, reaching significantly higher levels than in those with mild disease only at TP4 (p = 0.0408). Conversely, the EM subset was significantly more abundant in patients with severe disease at early time points, including TP1 (p = 0.0131) and TP2 (p = 0.0006) (Fig. 2D).

We further evaluated the phenotypic characteristics of SARS-CoV-2-specific CD8^+^ T-cells by performing dimensionality reduction with uniform manifold approximation projection (UMAP)^32^ and unsupervised clustering of multimer ^+^ CD8^+^ T-cells using the FlowSOM algorithm.^33^ UMAP showed distinct distributions of the SARS-CoV-2 multimer-reactive T-cells between patients with severe and mild COVID-19, highlighting different expression profiles of T-cell memory and activation markers (Fig. S3A). A heatmap generated by FlowSOM displayed the relative mean fluorescence intensity (MFI) for each marker across each clustered population (Fig. 2E and F). This unbiased clustering analyses further confirmed enrichment of T-cells with EM phenotype and activation markers in patients with severe disease, demonstrated by significant enrichment of population 2 at TP1 (p = 0.0069) and TP2 (p = 0.0087). On the contrary, for mild disease patients, significant enrichment of population 5 was observed, representing a cell population with TEMRA/naïve phenotype and minimal activation signature (TP1, p = 0.0278; TP2, p = 0.0065) (Fig. 2F and G).

In summary, we observed higher frequencies of SARS-CoV-2-specific CD8^+^ T-cells in patients with severe COVID-19 with an effector memory and activation profile, whereas patients with mild disease showed lower frequency and early onset of a TEMRA-like effector profile.

### Higher frequency and long-term persistence of antigen-specific T-cells in patients with severe COVID-19

**Fig. 3:**
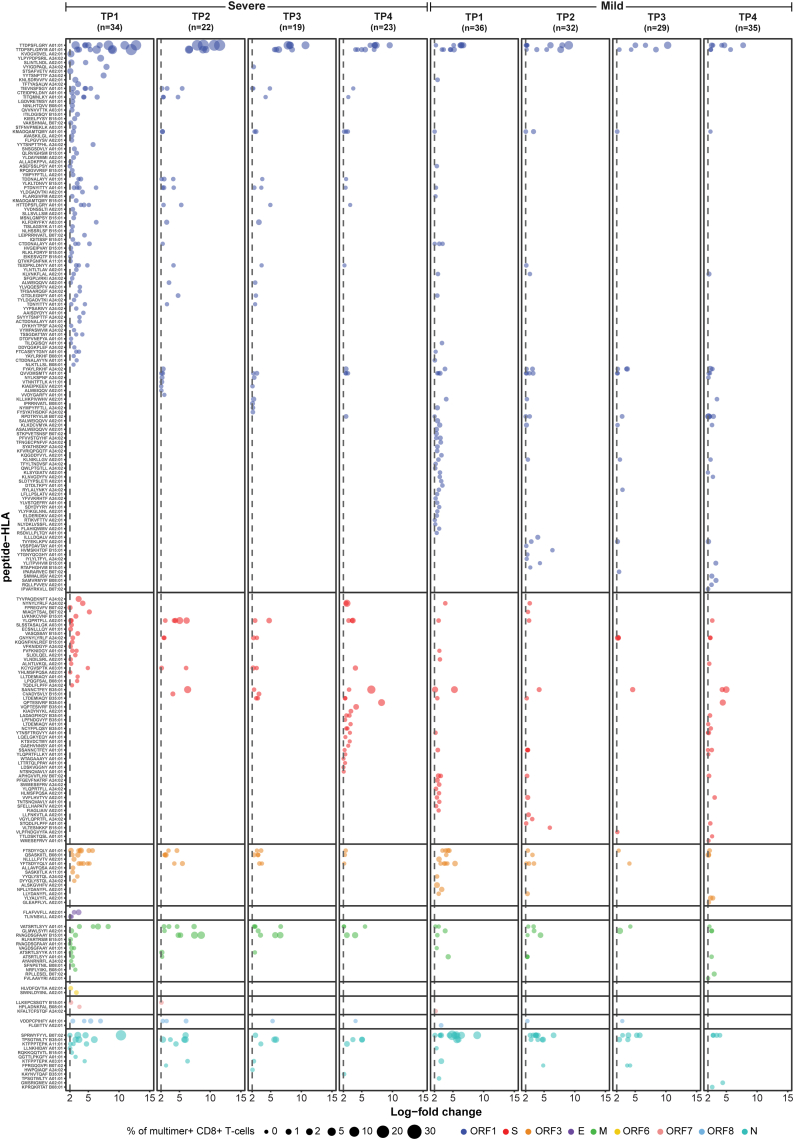
**Summary of SARS-CoV-2-specific T-cell responses in the patients with severe and mild COVID-19 at four time points.** A total 601 pHLA specificities were screened at TP1-TP4 using DNA-barcoded pHLA multimer assay, with an additional 357 Spike-specific pHLA included at TP4 for SARS-CoV-2 vaccinated patients. Numbers in parenthesis indicate the number of patients analysed for each time point and disease severity. Significant antigen-specific T-cell populations were identified based on the enrichment of DNA barcodes associated with each of the tested pHLA specificities (Log_2_ fold change ≥2 and p < 0.001, analysed using Barracoda). Only significant populations are shown in the plot. Each dot represents one peptide-HLA combination per patient and is coloured according to their protein of origin. Dot size is proportional to the estimated frequency calculated from the percentage read count of the associated barcode relative to the total CD8^+^ multimer^+^ T-cell population.

**Fig. 4:**
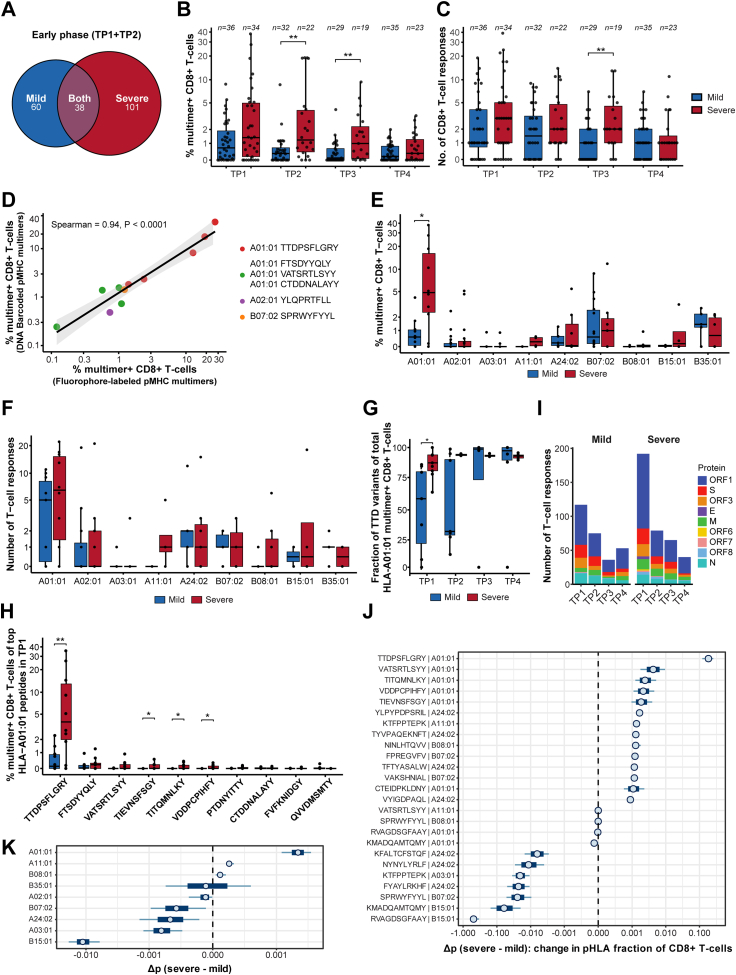
**SARS-CoV-2-specific CD8^+^ T-cell populations in patients with mild or severe COVID-19.** SARS-CoV-2–specific CD8^+^ T-cell populations were identified using DNA-barcoded pHLA multimer analysis. For each sample, number of epitope-specific populations and their frequency (% of CD8) restricted to different HLAs was calculated based on DNA-barcode analysis. (**A**) Venn diagram shows the number of unique SARS-CoV-2 epitopes identified in mild, severe, or both groups of COVID-19 patients during the early phase post-diagnosis (TP1 + TP2). (**B**, **C**) Box plots compare the frequency (% multimer^+^ CD8^+^ T-cells) (**B**) and the total number (**C**) of significant SARS-CoV-2-specific CD8^+^ T-cell populations between patients with severe and mild COVID-19 across the four time points (TP1-TP4). (**D**) Correlation between frequencies of multimer^+^ CD8^+^ T-cells measured using fluorochrome-labelled pHLA multimers and those obtained from DNA-barcoded pHLA multimer analysis for the selected immunodominant epitopes. pHLA multimers for FTS, VAT, and CTD were tested as a combined pool, hence plot reflects combined frequency of T-cells reactive to these three epitopes. Corresponding flow cytometry plots are shown in Fig. S3B. The black line indicates the linear regression, with the shaded grey area representing the 95% confidence interval. Spearman's rank correlation coefficient: ρ = 0.94, P < 0.0001. (**E**, **F**) Comparison of the sum of estimated frequency (%) (**E**) and the total number (**F**) of significant SARS-CoV-2-specific T-cell populations per HLA type at TP1 between patients with severe and mild COVID-19. (**G**) Box plot compares the fraction of the summed estimated frequency of TTDPSFLGRY (TTD) and its variant peptides (TTDPSFLGRYM and HTTDPSFLGRY) relative to the total HLA-A01:01-restricted peptide-specific T-cell populations in patients with mild and severe COVID-19. (**H**) Comparison of the estimated frequency of the top 10 HLA-A01:01-restricted epitopes, selected based on prevalence, between patients with mild and severe disease at TP1. TTDPSFLGRY and FTSDYYQLY also include the estimated frequency of their variant peptides (TTDPSFLGRYM and HTTDPSFLGRY for TTDPSFLGRY and YFTSDYYQLY for FTSDYYQLY). (**I**) Stacked bar plot summarises the number of antigen-specific T-cell populations derived from each SARS-CoV-2 protein across the four time points in cohorts of patients with mild and severe COVID-19. Statistical significance was calculated using Fisher's exact test (I) and Mann–Whitney test (B, C, E, F, G, H). Significance levels are indicated as ∗∗∗∗ (p < 0.0001), ∗∗∗ (p < 0.001), ∗∗ (p < 0.01) and ∗ (p ≤ 0.05). **(J, K)** Mixed-effect regression analysis identifies peptide–HLA pairs **(J)** and HLA alleles **(K)** associated with severity-related shifts in multimer-binding CD8^+^ T-cell frequencies. For each peptide–HLA pair or HLA allele, an average predictive comparison (AvgPC) was computed using a mixed-effects logistic regression model, quantifying the expected severity-associated change in the fraction of CD8^+^ T-cells binding the corresponding pHLA complex. For each testable observation, the model-predicted binding fraction was estimated assuming mild disease (p_0_) and severe disease (p_1_), while holding other predictors constant (patient identity, age, sex, peptide identity, HLA allele identity, and peptide–HLA pair, as applicable). The difference Δp = p_1_ − p_0_ was averaged across observations to obtain peptide–HLA pair–specific **(J)** or allele-specific **(K)** AvgPC values. Points indicate posterior medians, with thick and thin lines representing 50% and 90% credible intervals, respectively. In **(J)**, shown are the peptide–HLA pairs correspond to 20 selected peptides with the strongest evidence for positive or negative Δp, yielding 25 peptide–HLA pairs in total. The x-axis is displayed on a pseudo-logarithmic scale centred at zero to visualise both small and large effects.

To assess the breadth and frequency of antigen-specific CD8^+^ T-cell populations in mild and severe COVID-19, we characterised the epitope specificity of multimer–reactive CD8^+^ T-cells. Each pHLA multimer was labelled with a unique DNA barcode, allowing identification of individual epitope-specificity (log_2_ fold change >2; p < 0.001) and quantification of their frequencies within the total multimer-reactive CD8^+^ T-cell pool (Fig. 3). At TP1, SARS-CoV-2–specific CD8^+^ T-cells recognising at least one SARS-CoV-2 epitope included in the study were detectable in 76.5% (26 of 34) of patients with severe disease and 75.0% (27 of 36) of patients with mild disease. At the late time point (TP4), SARS-CoV-2–specific CD8^+^ T-cell populations were detectable in 65.2% (15 of 23) of patients with severe disease and 62.9% (22 of 35) of patients with mild disease, indicating sustained long-term persistence of SARS-CoV-2–specific CD8^+^ T-cells over time (Table S13). In total, we identified T-cell populations binding to 215 pHLA complexes, across all time points and patients, corresponding to 210 unique SARS-CoV-2 T-cell epitopes across the 9 analysed HLAs and 9 analysed proteins (Fig. 3, Table S14). Interestingly, in the early phase (TP1 and TP2), T-cell populations were detected against 139 and 98 epitope specificities in severe and mild COVID-19 patients, respectively, with only 38 specificities shared between the two groups, showing a broader repertoire of responses in the patients with severe COVID-19 (Figs. 3 and 4A).

The frequency (% multimer^+^ CD8^+^ T-cells) of antigen-specific T-cells and the number of unique T-cell populations were higher in patients with severe COVID-19 (Fig. 4B and C). In the acute phase of infection, patients with severe disease had a median frequency of total antigen-specific CD8^+^ T-cells of 1.4% (IQR 0.2–5.0). In contrast, in the patients with mild disease, it was 0.7% (IQR 0–1.9) (Fig. 4B, Table S13). Similarly, the median number of antigen-specific T-cell populations per patient was 3 (IQR 1–5) in patients with severe disease and 1 (IQR 0.8–4) in patients with mild disease (Fig. 4C, Table S13). These differences are unlikely due to differential number of tested pHLAs, as both patient groups were represented with similar HLA and epitope coverage (Fig. 1E).

The frequency of total antigen-specific CD8^+^ T-cells and the number of antigen-specific T-cell populations per patient gradually declined over subsequent months but remained higher for the patients with severe disease compared to the patients with mild disease throughout the investigated timespan (Fig. 4B and C, Table S13). Generally, patients with mild COVID-19 showed a faster contraction of CD8^+^ T-cell populations than those with severe disease, with the most significant differences in T-cell frequencies between the two groups observed at TP2 (p = 0.0025) and TP3 (p = 0.0079) (Fig. 4B), while differences in the number of antigen-specific T-cell populations were also most evident at TP3 (p = 0.01) (Fig. 4C). No significant differences were observed at TP4. Altogether, longitudinal profiling of T-cells revealed stronger and broader CD8^+^ T-cell activation and long-term persistence in patients with severe COVID-19.

### Severity-associated enrichment of epitope-specific CD8^+^ T-cells

As significantly higher frequencies of CD8^+^ T-cells were identified in patients with severe COVID-19 patients, we next investigated their association with HLA and epitope specificity. DNA-barcoded pHLA multimers identified several immunodominant epitope-specific CD8^+^ T-cell populations, with individual epitope-specific frequencies ranging from 0.1% to 28% of total CD8^+^ T-cells in individual samples. Using conventional single or dual fluorophore-labelled pHLA multimers, we validated these frequencies in samples from the two patient groups and found a strong correlation between the frequencies identified by the two approaches (Spearman's rank correlation coefficient; ρ = 0.94, P < 0.0001; Fig. 4D, Fig. S3B).

Despite the equal distribution of HLA-A∗01:01 in mild and severe patient groups (Fig. S4A), HLA-A∗01:01-specific T-cell frequencies were significantly higher in patients with severe disease compared to those with mild disease, and these represent the highest T-cell frequencies observed across all HLA restriction investigated, reaching up to 38% in individual patients (median 4.84% (IQR 2.45–16.58)) in the early phase of infection (TP1). In contrast, the maximum frequency of A∗01:01-specific T-cells in patients with mild COVID-19 was only 4.1% (median 0.58% (IQR 0.07–1.04)) (Fig. 4E, Fig. S4B, Table S15). Statistical comparison across all the nine HLAs for the frequency of T-cells showed significant enrichment of HLA-A∗01:01-specificities in the severe COVID-19 group (TP1, p = 0.015; TP2, p = 0.007; TP3, p = 0.012; TP4, p = 0.005) (Fig. 4E, Fig. S4B, Table S15). Similarly, the number of HLA-A∗01:01-restricted immunogenic epitopes was also higher for the patients with severe disease (TP1, p = ns; TP2, p = 0.013; TP3, p = 0.023; TP4, p = 0.009) (Fig. 4F, Fig. S4C, Table S15). Furthermore, the total percentage of estimated frequency and total number of HLA-A∗01:01-restricted immunogenic epitopes (normalised to the number of patients per HLA) was highest compared to all other HLAs in the severe group (Fig. S4D and E). Among the HLA-A∗01:01-restricted T-cell populations, the ORF1-derived TTDPSFLGRY peptide (including its variants TTDPSFLGRYM and HTTDPSFLGRY) was the most dominant epitope, exhibiting the highest antigen-specific T-cell frequency in both patients with mild and severe disease (Fig. 4G and H). However, the frequency of TTDPSFLGRY-specific T-cells (including its variants) was nearly tenfold higher in patients with severe COVID-19 (median 3.98% (IQR 2–13.14)) than in those with mild COVID-19 (median 0.14% (IQR 0–0.87)) at TP1 (p = 0.0191) (Fig. 4H). We also compared the impact of different SARS-CoV-2 proteins on the antigen-specific T-cell populations between patients with mild and severe COVID-19 and found no difference in the number of detected populations across all time points (Fig. 4I, Table S16).

Next, to test whether severe COVID-19 is associated with a different pattern of epitope-specific CD8^+^ T-cell reactivity than mild disease, we fitted a mixed-effects logistic regression model to the T-cell response data. The aim of the model was to predict the fraction of CD8^+^ T-cells binding to a given pHLA multimer (i.e., the epitope-specific CD8^+^ T-cell fraction). The model contained additive terms for peptide and HLA allele, allowing us to estimate how these factors relate to T-cell populations. It also included a peptide-HLA pair term to capture pair-specific deviations from the additive peptide and HLA effects. To account for potential confounding and between-patient heterogeneity, we included age and sex as covariates and a patient-specific random intercept. For each testable observation, we used the fitted model parameters to predict the expected epitope-specific CD8^+^ T-cell fraction. Specifically, we kept all predictors at their observed values, except for the severity indicator which we varied to compute a predicted CD8^+^ T-cell fraction under both mild and severe disease (*p*_mild_ and *p*_severe_, respectively). We defined Δ*p* = *p*_severe_-*p*_mild_ and then averaged Δ*p* across observations for each peptide-HLA pair to compute average predictive comparisons (AvgPCs),^39^ summarising severity-associated changes on an interpretable scale. We computed Δ*p* for each posterior draw and then averaged across observations to obtain posterior distributions of AvgPCs.

This analysis identified a set of peptide-HLA pairs with strong evidence of severity-associated changes in the epitope-specific CD8^+^ T-cells (Table S17). The most prominent positive shift is the HLA-A01:01–restricted ORF1ab epitope TTDPSFLGRY in severe disease, which shows a markedly larger effect size than other pairs on this scale. In addition, several other HLA-A01:01-restricted epitopes, similar to the one identified in frequency comparison (Fig. 4H), showed a strong shift in severe disease (Fig. 4J). Conversely, several pairs show strong evidence of higher epitope-specific CD8^+^ T-cell frequency in mild disease, including epitopes restricted to HLA-B15:01 (e.g. RVAGDSGFAAY and KMADQAMTQMY), HLA-A24:02 (FYAYLRKHF), and HLA-B07:02 (SPRWYFYYL) (Fig. 4J). For SPRWYFYYL this is consistent with both the observed higher frequency of specific T-cells (Fig. S4F, Table S14) and the greater prevalence of HLA-B∗07:02 donors in the mild group (37.8%; 14/37) than in the severe group (19.4%; 7/36) (Table S3) and remains supported after adjustment for potential confounders. Indeed, a strong correlation between the HLA-B∗07:02 restricted SPRWYFYYL–specific T-cell populations and mild disease, possibly due to cross-reactivity with the highly similar OC43/HKU-1-CoV N105–113 peptide (LPRWYFYYL), has been shown previously.^56^ At the allele level (averaging Δp across all testable observations involving each HLA allele), HLA-A∗01:01 shows the strongest evidence for an increase in severe disease, consistent with the pair-level dominance of A01:01-restricted epitopes. In contrast, HLA-B15:01 shows a clear decrease (higher in mild), also aligning with prior reports that B15:01 is associated with asymptomatic or mild COVID-19 (Fig. 4K).^57^ As sensitivity analyses, we refitted the mixed-effects regression model after excluding the two asymptomatic mild patients, and separately with adjustment for three broad comorbidity classes; severity-associated estimates were essentially unchanged in both the cases (Fig. S5).

In summary, we identify differential and selective expansion of CD8^+^ T-cells linked with immunodominant epitopes and HLA-specificity in severe and mild COVID-19.

### COVID-19 vaccination broadens T-cell repertoire and boosts long-term memory

We next evaluated the impact of vaccine-induced hybrid immunity on T-cell repertoire in 17 patients (severe, n = 10; mild, n = 7) who received either one or two doses of a COVID-19 vaccine between TP3 and TP4 (Table S2, Fig. S1A, Fig. S1D). Post-vaccination samples (TP4) of these patients were analysed using an additional 357 Spike-derived pHLA multimer pairs, covering the entire SARS-CoV-2 Spike protein (GenBank ID: QHD43416.1), encoded by the Pfizer-BioNTech BNT162b2 mRNA^58^ and the Moderna mRNA-1273^59^ vaccines (Table S6).

**Fig. 5:**
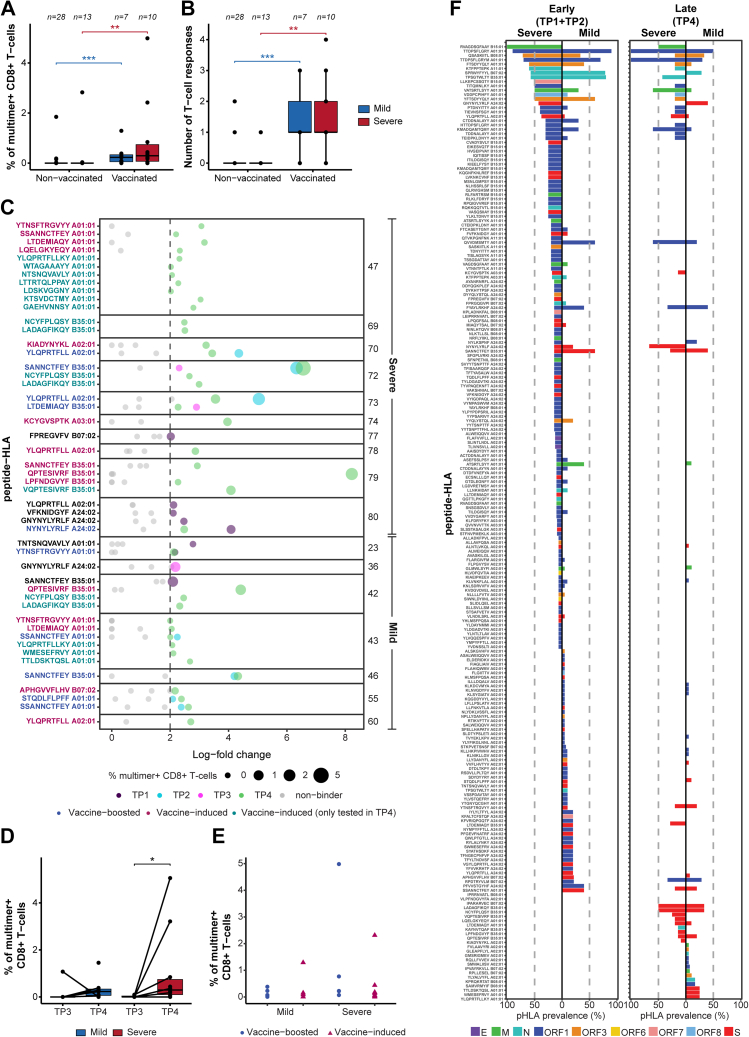
**Analysis of SARS-CoV-2 antigen-specific T-cell immunodominance and vaccine-driven hybrid immunity in patients with mild and severe COVID-19.** SARS-CoV-2–specific CD8^+^ T-cell populations were quantified using DNA-barcoded pHLA multimers across longitudinal time points, including post-infection vaccination follow-up in a subset of patients. (**A**, **B**) Box plot comparing the sum of the estimated frequencies (**A**) and the number (**B**) of SARS-CoV-2 Spike-specific T-cell populations at TP4 between patients with mild and severe disease and between vaccinated and non-vaccinated groups. Additional Spike peptides tested only in vaccinated patients at TP4 were excluded. (**C**) Summary of SARS-CoV-2 Spike-specific T-cell populations across four time points in the 17 vaccinated patients. Significant antigen-specific populations to individual pHLA pairs are coloured based on the time point at which they were identified and separated by Patient ID on the *y*-axis. Grey dots represent peptides without significant enrichment. Peptide-HLA labels on the *y*-axis are colour-coded to indicate the population classification at TP4: blue, vaccine-boosted; pink, vaccine-induced; turquoise, vaccine-induced populations tested only at TP4; and black, populations detected at other time points but not at TP4. (**D**) Box plot comparing the sum of the estimated frequencies of SARS-CoV-2 Spike-specific T-cell populations between mild and severe vaccinated individuals pre- (TP3) and post- (TP4) vaccination. (**E**) Estimated frequencies of SARS-CoV-2 Spike-specific T-cell populations identified post-vaccination in patients with mild and severe COVID-19. Only Spike pHLAs (n = 149 pHLA) analysed during the complete longitudinal analysis are included in this plot. (**F**) Prevalence according to disease severity of CD8^+^ T-cell recognition towards SARS-CoV-2 epitopes detected in early (TP1+TP2) and late (TP4) time points. Late prevalence includes T-cell populations recognising additional Spike peptides included only at T4. Only pMHC tested in more than 2 donors were included in this analysis. A dotted line is placed at 50% of prevalence to distinguish immunodominant epitopes. Bars are coloured according to their protein of origin. (A, B, D) Mann–Whitney test, ∗∗∗∗ (p < 0.0001), ∗∗∗ (p < 0.001), ∗∗ (p < 0.01) and ∗ (p ≤ 0.05).

Both the frequency and the number of Spike-specific T-cell populations were significantly increased in vaccinated compared to non-vaccinated individuals at TP4 (frequencies: mild p = 0.00027, severe p = 0.0036; number of populations: mild p = 0.00020, severe p = 0.0016) (Fig. 5A and B). Overall, vaccination either enhanced the frequencies of infection-induced Spike-specific T-cells or induced de novo vaccine-specific T-cell populations (Fig. 5C). These results are consistent with previous reports demonstrating a broader repertoire of Spike-specific CD8^+^ T-cell populations following vaccination compared with natural infection, including vaccine-preferred epitopes detected exclusively in vaccinated individuals.^60^, ^61^, ^62^ Furthermore, post-vaccination analysis of the additional Spike peptide library (tested only at TP4) identified T-cell reactivity to 12 Spike pHLA complexes across all vaccinated patients (Fig. 5C). Notably, even though the frequency of Spike-specific T-cells was significantly increased only in the severe COVID-19 group post-vaccination compared to pre-vaccination (p = 0.0132) (Fig. 5D), both patient groups were efficient in enhancing pre-existing Spike-specific T-cell populations or mounting de novo vaccine-specific T-cell populations following vaccination (Fig. 5E). These results show that vaccination (post-infection) further boosts existing antigen-specific T-cell populations as well as broaden the overall CD8^+^ T-cell repertoire irrespective to the outcome of the SARS-CoV-2 infection.

### Immunodominant SARS-CoV-2 epitopes establish long-term CD8^+^ T-cell memory

We next evaluated the long-term persistence and prevalence of epitope-specific T-cells across the two patient groups. For robustness, only epitopes tested in at least three individuals with the corresponding HLA molecule per time point and severity group were analysed (Fig. 5F, Fig. S6, Table S18). In the early phase following infection (TP1 + TP2), T-cell recognising several highly prevalent epitopes (prevalence ≥50%) were detected both in both patients with severe and mild COVID-19. Among the severe COVID-19 group, eight epitopes were determined as immunodominant, based on T-cell recognition in more than 50% of the tested samples and detection in at least three or more patients. In patients with mild COVID-19, seven immunodominant epitopes were identified. The epitopes TTDPSFLGRY (and its variant TTDPSFLGRYM; HLA-A∗01:01), SPRWYFYYL (HLA-B∗07:02), and TPSGTWLTY (HLA-B∗35:01) were considered immunodominant in both groups. Furthermore, patients with severe COVID-19 exhibited a broader repertoire of SARS-CoV-2-specific T-cell epitopes and a higher prevalence of T-cell recognition for individual epitopes (Fig. 5F).

At TP4 (approximately 220 days post-infection, including the post-vaccination analysis), long-term memory was observed to have a more selective repertoire, primarily consisting of epitopes that were highly prevalent during the acute phase, along with the vaccine-boosted or vaccine-driven T-cell populations (Fig. 5F). Altogether, the long-term CD8^+^ T-cell memory was established against 43 and 47 epitopes in patients with severe and mild disease, respectively. The long-term immunodominance was found for six of these epitopes in the severe COVID-19 group with TTDPSFLGRY (and its variant TTDPSFLGRYM; HLA-A∗01:01 restricted) being the most dominant antigen-specific T-cell population with 100% prevalence. In the mild patient group, none of the antigen-specific T-cells showed immunodominance (prevalence ≥50%), with TTDPSFLGRY-specific T-cells having a 50% prevalence in this group (Table S18).

In summary, long-term SARS-CoV-2-specific CD8^+^ T-cell memory is directed towards a few immunodominant epitopes established in the early phase of infection and further broadened by COVID-19 vaccination.

### Single-cell analysis reveals enhanced cytotoxic T-cell profile in mild COVID-19

**Fig. 6:**
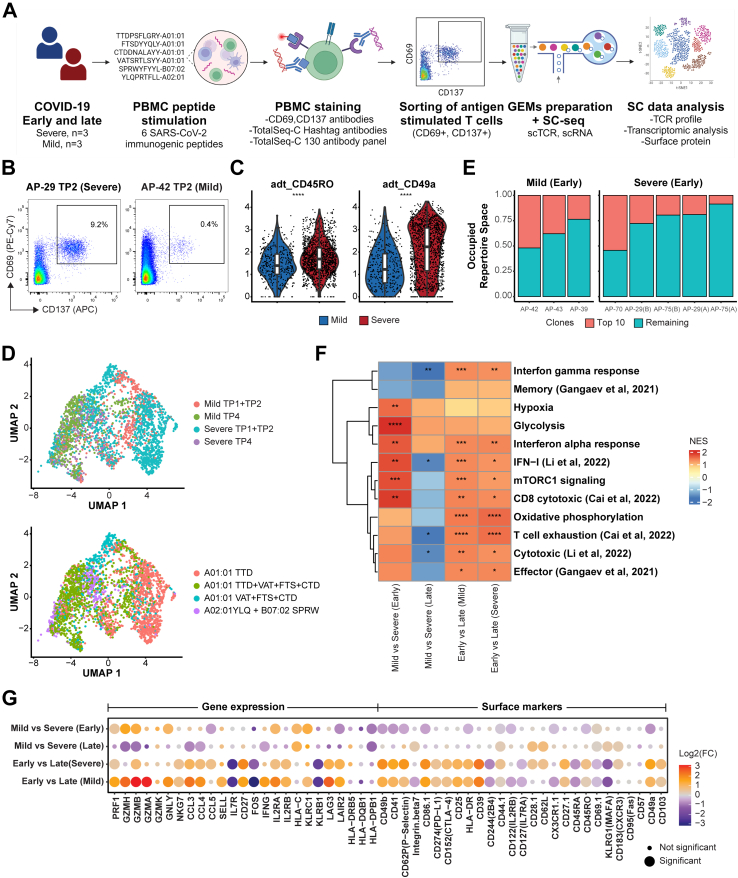
**Single-cell analysis comparing antigen-specific CD8^+^ T-cells of patients with mild and severe COVID-19.** (**A**) Experimental pipeline for single-cell transcriptome, surface proteome, and TCR analysis of three patients with mild and three patients with severe COVID-19, analysed during the early and late phase of infection. PBMCs were stimulated with SARS-CoV-2-derived peptides for 24 h, and activated CD8^+^ T-cells were sorted based on CD69 and CD137 expression. Sorted cells were processed using the 10x Genomics 5′ v2 chemistry for downstream single-cell analysis. (**B**) Representative flow cytometry plots showing antigen-stimulated CD8^+^ T-cells sorted for single-cell analysis based on CD69 and CD137 expression. (**C**) Comparison of surface markers expression levels between severe and mild samples for early time point. Mann–Whitney test: ∗p < 0.05, ∗∗p < 0.01, ∗∗∗p < 0.001, ∗∗∗∗p < 0.0001. (**D**) UMAP visualisation of scRNA-seq data coloured by a combination of severity and time point (**top**) and by epitope specificity (**bottom**). A total of 2762 CD8^+^ T-cells were included for early/late phase single-cell analysis (**D**–**G**). (**E**) TCR clonal composition within early (TP1/TP2) samples for mild and severe conditions. Patient ID labels (*x*-axis) are described in Table S10. TCR clones are merged according to proportion into two categories: the top 10 represented clones and the remaining ones. (**F**) Gene set enrichment analysis (GSEA) for selected gene sets. Heatmap of normalised enrichment score (NES) coupled with adjusted p-values (∗p < 0.05, ∗∗p < 0.01, ∗∗∗p < 0.001, ∗∗∗∗p < 0.0001). (**G**) Selected genes and surface markers (columns) identified from differential expression analysis between cells grouped by severity and time point (rows). Significant defined by adjusted p-value <0.05.

As disease severity in COVID-19 was associated with differences in the frequency and phenotype of T-cells, we combined activation-induced marker (AIM) assays and single-cell analysis to investigate the functional differences of T-cells across severity groups. We integrated transcriptomic profiling, oligo-tagged antibody staining (CITE-seq), and T-cell receptor sequencing (TCR-seq), analysing samples from six patients (mild, n = 3; severe, n = 3) at early (TP1 or TP2) and late (TP4) time points (Fig. 6A). From our pHLA multimer analysis, we selected six epitopes; HLA-A01:01–restricted TTDPSFLGRY, FTSDYYQLY, VATSRTLSYY, and CTDDNALAYY (mild, n = 3; severe, n = 3); HLA-B07:02–restricted SPRWYFYYL (severe, n = 1); and HLA-A∗02:01–restricted YLQPRTFLL (severe, n = 1), detected in these patients at both early and late timepoints. Peripheral blood mononuclear cells (PBMCs) were stimulated with either individual peptides or an HLA-matched peptide pool for 24 h (Table S10), and cells were sorted for single-cell analysis based on the expression of activation markers CD69 and CD137 (Fig. 6B, Fig. S7). After demultiplexing and quality control filtering, 2784 singlet cells were retained for downstream analyses (Figs. S8 and S9).

At early time points, T-cells of patients with severe COVID-19 showed higher expression of cell surface markers (CITE-seq) associated with antigen-specific memory markers (CD44, CD122, CX3CR1, CD45RO), and tissue-resident marker CD49a, whereas T-cells of the patients with mild COVID-19 showed enhanced expression of costimulatory molecule CD28 and the resident memory marker CD69 (Fig. 6C, Fig. S10A). On the contrary, at the late time point (about six months post-infection), T-cells of patients with mild COVID-19 showed higher expression of activated memory T-cells; CD44, CD62L, CD28, and KLRG1 (Fig. S10B).

The UMAP distribution of transcriptomic data revealed distinct clustering of T-cells from patients with mild and severe COVID-19 at early time points, which converged at the late time point, suggesting that COVID-19 severity is associated with differential gene expression during acute SARS-CoV-2 infection (Fig. 6D). These clustering differences were not attributable to T-cell receptor (TCR) clonal distribution or to the overall TCR repertoire space occupied by expanded clones (top 10 TCR clones per sample) in patients with mild versus severe COVID-19 (Fig. 6E). However, gene set enrichment analysis of multiple T-cell activation and function–related pathways^44^^,^^46^, ^47^, ^48^ revealed that at early time points, T-cells from patients with mild COVID-19 exhibited significantly stronger activation signatures than those from severe disease, including pathways associated with CD8^+^ T-cell cytotoxicity and interferon (IFN) responses. Additional pathways, such as mTORC1 signalling, glycolysis, and hypoxia, were also significantly enriched, consistent with higher activation of cytotoxic gene programs in CD8^+^ T-cells from patients with mild COVID-19 during early infection.^63^, ^64^, ^65^, ^66^ By the late time point, these differences had diminished, and T-cells from patients with severe COVID-19 displayed comparable or even higher activation profiles. Moreover, within both severity groups, early-phase T-cells showed significantly stronger activation than late-phase T-cells across many of these pathways (Fig. 6F, Fig. S11).

To further investigate the elevated genes associated with more efficient antiviral effector response in mild disease, we performed differential gene expression analysis at both the single-cell and pseudobulk levels (Fig. S11). In line with the pathway-level findings, T-cells from patients with mild COVID-19 at early time points displayed significantly higher expression of key effector and cytotoxic genes, including perforin (*PRF1*), granzymes (*GZMH, GZMB*), and granulysin (*GNLY*). These results suggest that during the acute phase of infection, mild disease is associated with a more robust cytotoxic program. In contrast, at late time point these genes were more highly expressed in patients with severe COVID-19 (Fig. 6G). Furthermore, the dominance of cytotoxic signatures at early versus late time points was much stronger in patients with mild COVID-19 (Fig. 6G). At the protein level, cell surface expression of activation markers CD49b, CD41, CD39, and CD69 supports the activation profile observed at the transcriptomic level for T-cell activation at early time points in both mild and severe COVID-19, however, fails to capture the dynamics of differential activation of T-cells between disease severities (Fig. 6G).

In summary, our single-cell analysis identifies reduced expression of genes associated with CD8^+^ T-cells activation and cytotoxicity in patients with severe COVID-19 during the acute phase of SARS-CoV-2 infection.

### Immunodominant antigen-specific T-cells are differentially activated in patients with mild and severe COVID-19

**Fig. 7:**
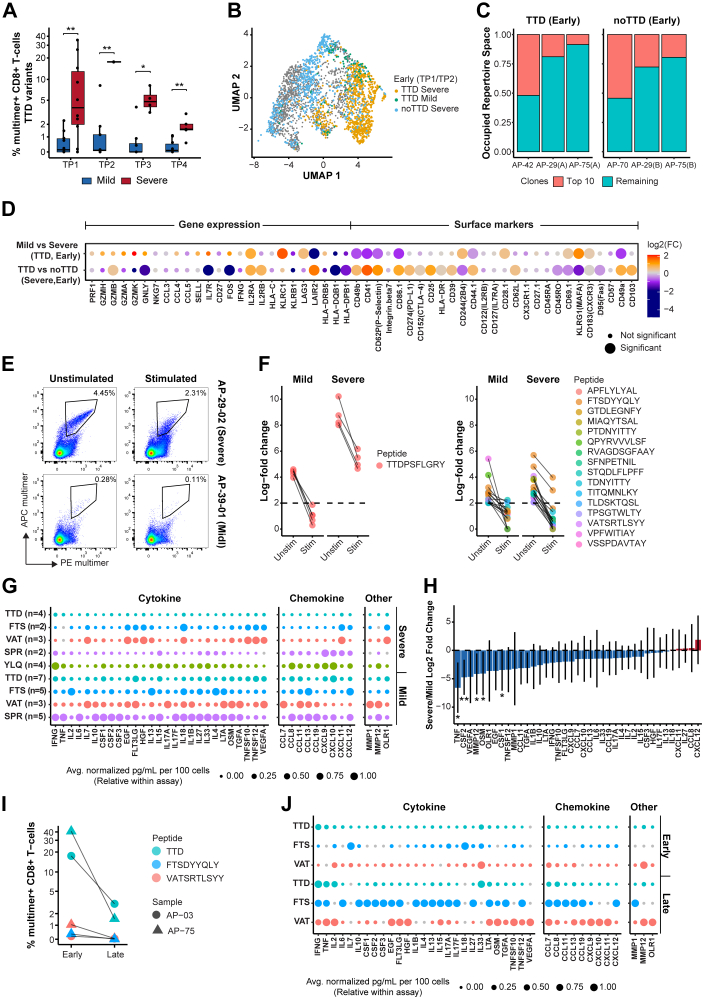
**Functional assessment of antigen-specific T-cells in patients with mild and severe COVID-19.** (**A**) Sum of estimated frequencies of HLA-A01:01-restricted TTD-specific T-cells (including its variants TTDPSFLGRYM and HTTDPSFLGRY) in patients with mild and severe COVID-19 across the different time points determined by DNA-barcoded pHLA analysis. Mann–Whitney test, mild vs severe; TP1 (p = 0.00685), TP2 (p = 0.00685), TP3 (p = 0.0107), TP4 (p = 0.00733). P-values were adjusted using the Bonferroni method and found non-significant when comparing across time points. (**B**) UMAP visualisation of scRNA-seq data for early (TP1/TP2) time points coloured by a combination of peptide stimulation groups (TTD and no TTD) and disease severity. Early phase single-cell analysis (**B**–**D**) includes 1523 CD8^+^ T-cells, comprising 1414 cells from patients with severe COVID-19 (840 TTD-specific and 574 non-TTD-specific) and 109 cells from patients with mild COVID-19, all of which were TTD-specific. (**C**) TCR clonal composition of TTD- and no TTD-specific T-cell populations within early-phase (TP1/TP2) samples, derived from single-cell TCR sequencing data. Clones are grouped according to proportion into two categories: the 10 most represented clones and the remaining clones. (**D**) Differential expression analysis of selected genes and surface markers based on single-cell transcriptomic and proteomic data, between cells grouped by disease severity and peptide specificity. Genes and markers with significant differences (adjusted p < 0.05) are indicated. (**E**) Representative flow cytometry dot plots of double-positive (PE^+^APC^+^) multimer-binding T-cell populations with and without peptide stimulation used to assess TCR down-regulation. Unstimulated controls were treated with equimolar DMSO. (**F**) Antigen-specific T-cells identified using pHLA multimers in peptide-stimulated samples compared to unstimulated controls in mild (n = 6) and severe (n = 4) patients. Only significant antigen-specific T-cell populations (Log_2_ fold change ≥2 and p < 0.001) identified in unstimulated controls were compared with the respective peptide-stimulated samples. **(G)** Mean protein secretion levels (pg/mL per 100 cells) of antigen-specific T-cells at the early phase (TP1/TP2) in patients with severe (n = 9) and mild (n = 9) COVID-19 following 24-h peptide stimulation, measured using Olink assay. Values were scaled to a range of 0–1. (**H**) Log_2_ fold change in protein secretion levels between severe and mild groups during the early phase. Error bars indicate 95% confidence intervals. Mann–Whitney test; ∗∗p < 0.01, ∗p < 0.05. (**I**) Estimated frequency (%) of TTD-specific T-cells in patients with severe COVID-19 at early and late phases post-diagnosis. **(J)** Magnitude of mean protein secretion levels (pg/mL per 100 cells) for each peptide, calculated as the average for all samples within each phase (early and late), following peptide stimulation of samples from patients with severe COVID-19 shown in (I). Values were scaled to a range of 0–1. In (C and D), TTD refers to TTDPSFLGRY-specific T-cells, and no TTD refers to T-cells specific to other epitopes (FTSDYYQLY, VATSRTLSYY, CTDDNALAYY, YLQPRTFLL, or SPRWYFYYL). Abbreviations: TTD, TTDPSFLGRY; FTS, FTSDYYQLY; VAT, VATSRTLSYY; CTD, CTDDNALAYY; YLQ, YLQPRTFLL; SPR, SPRWYFYYL.

As we observed both a substantially higher frequency of SARS-CoV-2-specific T-cells in patients with severe COVID-19 (Fig. 4) and a reduced activation profile of T-cells identified in the single-cell analysis (Fig. 6), we next investigated the relationship between epitope specificity and differential functional profiles of T-cells in mild and severe disease. We focused the analysis on HLA-A∗01:01-restrcited TTDPSFLGRY (TTD)-specific T-cells. TTD peptide has been identified as the most immunodominant SARS-CoV-2 epitope by us and others.^19^^,^^47^^,^^67^ It establishes long-term memory in both patients with severe and mild COVID-19 (Fig. 5F and Table S14) and induces significantly higher proportion of T-cells in the severe COVID-19 group across all timepoints (TP1 p = 0.0069; TP2 p = 0.0069; TP3 p = 0.0107; TP4 p = 0.0073) (Fig. 7A).

In the single-cell analysis during early infection (TP1/TP2), TTD-specific T-cells from patients with severe COVID-19 clustered distinctly from both patients with mild disease and non-TTD-specific T-cells, indicating a unique transcriptional activation state (Fig. 7B). This difference was independent of TCR clonality (Fig. 7C). Severe TTD-specific cells showed lower expression of cytotoxic genes (*PRF1, GNLY, GZMH, GZMA, GZMB, GZMK*) compared with patients with mild disease, while non-TTD-specific T-cells within the COVID-19 severe group exhibited higher cytotoxic gene expression, including significantly elevated *GNLY*, suggesting reduced cytotoxic potential of TTD-specific cells. In contrast, severe TTD-specific cells upregulated interferon signalling–associated genes (*GNZB, IL2RA, UL2RB*), reflecting a more inflammatory-biased transcriptional program (Fig. 7D, Fig. S12A).

In patients with mild disease, TTD-specific T-cells expressed *IL2, LAG3*, and *KLRC1* and elevated cell-surface levels of activation and tissue-homing markers (CD69, KLRG1, CD28, CXCR3, CD49a/b, CD103, CD62 P/L, CD44), indicating they are transcriptionally poised for efficient cytotoxic and tissue-localised responses. Gene enrichment analysis revealed upregulated glycolysis and hypoxia pathways in mild TTD-specific cells, whereas in patients with severe disease these pathways were enriched in non-TTD T-cells, suggesting metabolic dysregulation of immunodominant TTD-specific cells in severe disease. Supporting this, *UCP2* was selectively upregulated in TTD-specific T-cells from patients with severe COVID-19, a gene known to limit glycolysis and control T-cell differentiation (Fig. S11B and D).^68^ Next, to understand the role of TCR composition, we evaluated antigen-specific TCR gene segments for any preferential enrichment. We observed a higher V9-2 and V27 gene segment usage for alpha and beta chains, respectively, in TTD-specific cells compared to no TTD cells (Fig. S13). Furthermore, an antigen-specific combination of different gene segments was evident in both alpha and beta chains across the evaluated T-cell specificities (Fig. S14).

Together, these findings indicate that in patients with severe COVID-19, immunodominant TTD-specific CD8^+^ T-cells display signs of metabolic and functional dysregulation, including altered activation and reduced cytotoxic gene expression profiles.

### T-cells from patients with severe COVID-19 show reduced TCR responsiveness and cytokine production in the acute phase of infection

Based on the single-cell data highlighting altered activation of SARS-CoV-2-specific CD8^+^ T-cells from patients with severe COVID-19, we next sought to functionally validate these observations. First, we assessed T-cell responsiveness using a TCR down-regulation assay, which measures antigen-induced internalisation of surface TCRs as a proxy for activation. Antigen-induced T-cell activation leads to TCR down-regulation and thus reduces the availability of cell-surface TCRs for pHLA multimer-based T-cell detection.^27^ Utilising this approach, we used DNA-barcoded pHLA multimers to measure T-cell activation by comparing the level of TCR-down regulation (inversely proportional to the pHLA multimer binding) with and without antigen stimulation.

Seventeen epitope-specific T-cell responses were analysed in PBMCs from ten patients at TP1/TP2, four of which were also included in the single-cell analysis. After 24-h peptide stimulation (Table S9), samples from patients with mild COVID-19 (n = 6) showed near-complete TCR internalisation, whereas samples from patients with severe COVID-19 (n = 4) retained substantial pHLA multimer binding, indicating T-cells that do not, or only partially respond to antigen challenge (Fig. 7E, Fig. S15A). Furthermore, changes in MFI levels were comparable between mild and severe samples, suggesting no measurable difference in cell-surface TCR density (Fig. S15B). DNA Barcode-based quantification of individual pHLA-specificities confirmed incomplete TCR down-regulation (log_2_FC > 2) in TTD-specific T-cells from all patients with severe COVID-19, while TTD and other antigen-specific T-cells from patients with mild disease showed complete internalisation (Fig. 7F). Another epitope, FTSDYYQLY (HLA-A∗01:01-restricted), also showed similar incomplete TCR down-regulation in two patients with severe disease (Fig. 7F, right).

To further assess the functional capacity of epitope-specific T-cells in patients with mild and severe COVID-19, we used an Olink assay panel to measure 45 different cytokines and chemokines after stimulating T-cells for 24 h with individual epitopes in 18 patients (mild, n = 9; severe, n = 9) (Table S11). In the acute phase of infection, epitope-specific T-cells from patients with severe COVID-19 exhibited an overall reduced cytokine and chemokine response compared to those with mild disease, with lower levels observed for the majority of analytes measured. Specifically, T-cells from patients with severe disease showed significantly reduced secretion of TNF-α (p = 0.0130), CSF1 (CSF; p = 0.0468), CSF2 (GM-CSF; p = 0.0094), and MMP12 (p = 0.0313), as well as reduced levels of key effector cytokines including IFN-γ, IL-2, and IL-6, indicating a broader and polyfunctional effector profile of CD8^+^ T-cells in patients with mild disease compared with those from patients with severe disease (Fig. 7G and H). At the individual epitope level, TTD-specific CD8^+^ T-cells from patients with severe disease (n = 4) showed a lower overall cytokine and chemokine secretion profile compared to TTD- (n = 7) and SPR-specific (n = 5) cells from patients with mild disease (Fig. S16A and B). Furthermore, analysis of paired samples from two patients with severe disease with the highest frequency of SARS-CoV-2-specific T-cells, revealed up to eightfold higher cytokine secretion at late time points (∼6 months post-infection) compared to the acute phase, suggesting partial recovery of T-cell function over time (Fig. 7I and J, Fig. S16C).

In summary, functional assays revealed that SARS-CoV-2-specific CD8^+^ T-cells from patients with severe COVID-19 show reduced activation and cytokine secretion in response to antigen stimulation during the acute phase, aligning with transcriptional and metabolic signatures identified in the single-cell analyses.

## Discussion

Since the onset of the COVID-19 pandemic, multiple studies have underscored the essential role of CD8^+^ T-cells in initiating an early and effective antiviral immune response.^2^^,^^12^^,^^14^^,^^69^^,^^70^ Yet, the epitope-specific dynamics of CD8^+^ T-cell activation associated with disease severity, and their long-term immunological impact, remain incompletely defined. Here, we provide a comprehensive analysis of the breadth, frequency, and functional diversity of antigen-specific CD8^+^ T-cells across patients with mild and severe COVID-19. We demonstrate that the epitope-specific T-cell repertoire differs in both scale and phenotype between these groups. Precise monitoring of the frequency and phenotype of epitope-specific T-cell populations is crucial to understand their impact on disease outcome as well as in establishing long-term memory pool. By profiling 553 CD8^+^ T-cell epitopes across patients with severe and mild COVID-19, this study resolves T-cell dynamics across COVID-19 disease severity. Furthermore, following T-cells from acute infection until they establish long-term memory, we identify phenotypic and functional features of T-cells that may be crucial for viral disease outcomes.

Our data identifies several critical features of CD8^+^ T-cell kinetics in SARS-CoV-2 infection associated with COVID-19 disease. In the acute phase of infection, SARS-CoV-2-reactive CD8^+^ T-cell repertoire is driven by a large set of immunogenic epitopes. Only 38 of these epitopes overlapped in mild and severe disease, and the remaining were unique to the mild or severe groups. These observations further supported by a mixed-effects binomial logistic regression analysis showing a higher probability of T-cell populations specific to HLA-A01:01–restricted epitopes in severe disease, whereas HLA-B15:01– and HLA-B07:01–restricted epitopes were more frequently associated with mild disease. Although the mechanistic basis underlying the preferential expansion of specific epitopes in severe versus mild COVID-19 remains incompletely understood, similar patterns have been reported for individual epitopes in prior studies. For example, TTD epitope-specific (HLA-A01:01-restricted) CD8^+^ T-cells have been found at particularly high frequencies in patients with severe disease,^47^ whereas epitopes restricted to HLA-B15:01^57^ and HLA-B07:01^56^ have been found in asymptomatic SARS-COV-2 infection or mild disease. These observations suggest that composition of epitopes may be relevant to study in the context of T-cell immunity in viral infections.

Contrary to the acute phase, the long-term CD8^+^ T-cell memory (based on the TP4 data) is established against only a small fraction of T-cell epitopes. This may in part reflect assay sensitivity, as low-frequency memory populations could fall below the detection threshold, despite the high sensitivity of the DNA-barcoded pHLA multimer assay (detection limit ∼0.001% of CD8^+^ T-cells).^31^ Immunodominant epitopes with high prevalence (HLA-specific) in the acute phase contributed the most in establishing the long-term T-cell populations, likely due to the higher initial frequencies. In line with existing reports, COVID-19 vaccination boosted the T-cell frequency of the memory T-cells induced by primary infection.^71^, ^72^, ^73^ Importantly, vaccination also generated de novo antigen-specific T-cell populations in patients with severe and mild COVID-19, thus broadening the overall T-cell repertoire. Although we have not tested the T-cell immunity concerning the evolving landscape of SARS-CoV-2 variants,^74^ the long-term memory constituted largely by epitopes from non-Spike regions of SARS-CoV-2 is likely to provide better protection as compared to T-cells specific to only Spike epitopes against different variants with high mutation frequency in Spike protein.^75^

Previous studies have reported antigen- and HLA-specific association in asymptomatic or mild SARS-CoV-2 infection.^56^ Our data demonstrates a substantially high frequency of CD8^+^ T-cells and the number of distinct antigen-specific T-cell populations in patients with severe COVID-19 is strongly driven by HLA-A∗01:01-restricted antigens. In the early outbreak of the COVID-19 pandemic in Italy and later in different geographical locations, HLA-A∗01:01 populations have been found susceptible to severe COVID-19.^76^, ^77^, ^78^ Persistent antigen stimulation has been shown to impair cytotoxic T-cell function in viral infections and cancer.^79^ In SARS-CoV-2 this has been largely observed in patients with severe COVID-19 and could be due to prolonged viral encounters and antigen stimulation, also due to associated comorbidities and age.^80^^,^^81^ Similarly, analysis of alveolar T-cells in severe COVID-19 indicated ORF1ab-specific T-cells, compared to T-cells specific to structural proteins, associated with poor survival after hospitalisation, potentially due to delayed T-cell response or higher viral replication leading to expansion of ORF1ab-specific T-cells.^82^

In this study, ORF1-derived HLA-A∗01:01-restricted TTD antigen was the most immunodominant epitope in both mild and severe disease patients; however, the T-cell frequency was significantly higher in severe cases and constituted the majority of the total SARS-CoV-2 antigen-specific T-cells in individual patients. Our previous study has also shown reduced cytokine secretion by TTD-specific T-cells in patients with severe COVID-19.^19^ Similarly, another study also found impaired cytokine secretion by TTD-specific cells in patients with severe COVID-19.^47^ By comparing multiple antigen-specificities in single-cell analysis and cytokine profiling, we now show altered metabolic reprogramming associated with T-cell exhaustion and reduced cytokine secretion capacity of immunodominant epitope-specific T-cells in patients with severe COVID-19. Further, TCR down-regulation assay indicate that some of the high-frequency T-cells restricted to immunodominant epitopes such as TTD and FTS does not fully respond to antigen-stimulation and may contribute to the reduced cytokine and functional activation in severe disease. However, using our TCR down-regulation assay it was not possible to understand if this effect is driven by complete lack of functional responses only in a fraction of the epitope-specific T-cells or an overall partial reduction in TCR-response by all the epitope-specific T-cells. Altogether, these data suggests that immunodominance based on population frequency does not necessarily equate to protective efficacy and, in some contexts like severe COVID-19, may even mark a dysregulated or pathogenic response. Future work could explore whether such ‘dysfunctional immunodominance’ is a feature of other severe viral infections. To our knowledge, such large-scale expansion of antigen-specific T-cells has been observed in CMV infection^83^ and a comparative assessment of antigen-specific T-cells from SARS-CoV-2 and CMV infection in the acute phase would be relevant to gain further understanding of antigen-specific dynamics of T-cell activation and related functional consequences. In this regard, it would be also relevant to evaluate the frequency as well as the cytokine release capacity of epitope-specific T-cells at the site of infection such as upper airways and lungs to assess related tissue-specific impact. Furthermore, several factors can influence T-cell immunodominance and high-frequency expansions, including early antigen availability, antigen presentation efficiency, and frequency of naïve TCR repertoire.^84^ Further studies are needed to understand which of these features contribute to the immunodominance of TTD epitope and potential mechanism that drives such large-scale expansion specifically in severe COVID-19.

It is important to emphasise that the reduced function of T-cells in patients with severe COVID-19 seems to resolve over time as at the late time point T-cells from these patients have comparable levels of transcriptomic and cytokine profile in response to peptide stimulation. Together, we hypothesise that a lack of early viral control may lead to massive proliferation of CD8^+^ T-cells to counteract the persistent viral load in severe COVID-19. Pending viral control the SARS-CoV-2-specific CD8^+^ T-cell compartment is gradually reverting to their functional state. As such severe disease patients respond to vaccines at an equal level as patients with mild disease.

In contrast, early activation of cytotoxic T-cells in SARS-CoV-2 infection and vaccination has been shown previously and supports the importance of T-cells in early viral control.^7^^,^^85^^,^^86^ Compared to the higher proliferation observed in patients with severe COVID-19, those with mild disease showed early recruitment of memory T-cells with TEMRA-like features. It's possible that the differential T-cell phenotype reflects state of infection, especially as the phenotype in patients with mild disease indicate early viral clearance. Although, the viral load data (based on PCR-test at the time of enrolment) indicate inverse correlation with disease severity in this patient cohort. Existing data dissecting immune response in mild and asymptomatic SARS-CoV-2 infection support early activation of cytotoxic T-cells for effective viral clearance.^17^^,^^18^ T-cells specific to HLA-B∗07:02-specific immunodominant epitope SPRWYFYYL were most prevalent in mild disease group and also supported by our mixed-effects binomial regression model. We and others have shown the presence of HLA-B∗07:02-SPRWYFYYL T-cells in SARS-CoV-2 unexposed individuals and sequence homology between SPRWYFYYL peptides and peptides derived from other human coronaviruses (HCoVs)^19^^,^^87^ and it has been associated with mild cases.^56^ Thus, a pre-existing T-cell memory might be involved in rapid expansion and effective viral control in mild or asymptomatic patients.^2^^,^^67^^,^^88^^,^^89^ Indeed, our flow cytometry data support this notion, as we observe an early activation and TEMRA profile and patients with mild disease compared to those with severe disease, and we also detect a higher frequency of HLA-B∗07:02-SPRWYFYYL-specific T-cells in patients with mild disease. In future, identification of individual T-cell epitopes that induce early T-cell activation could be useful in determining immune memory as well as using such antigens for vaccine design.^90^

Our study has potential limitations. The results reflect the study population and might not be fully generalisable to more diverse global populations. The stratification of patients into mild and severe categories is largely based on hospitalisation status, with hospitalisation duration varying widely (1 day–36 days). The cohort size (36 patients with severe COVID-19 and 37 with mild disease) enabled comparable statistical analyses; however, subgroup analyses (including single-cell profiling and functional assays) were performed on selected samples based on detectable antigen-specific T-cell responses and sample availability, resulting in limited statistical power. These findings require validation in larger and more representative cohorts. Additionally, although sampling time points were broadly comparable between severity groups, longitudinal data on viral clearance and post-diagnosis viral load were not available. As a result, we could not directly evaluate the contribution of viral dynamics to the observed differences in antigen-specific CD8^+^ T-cell frequency and phenotype. While our study shows enhanced frequency of antigen-specific T-cell populations in severe COVID-19, the causal nature of this relationship remains to be established. Future studies, including prospective clinical investigations and mechanistic experiments in animal models, will be crucial to determine whether these T-cell responses contribute to disease severity or are a consequence of it. Furthermore, the severe COVID-19 group also included patients with comorbidities, which may influence COVID-19 disease severity and bias in T-cell immune response.^91^^,^^92^ Due to the limited cell number, our single-cell data lacked the power to follow individual TCR clones from the early phase to the memory phase for individual antigen-specificity to investigate clonal dynamics of CD8^+^ T-cells in relation to their functional characteristics.^93^ Also, single-cell data comparing TTD-specific T-cells with non-TTD-specific cells is limited by number of analysed samples, and the data may be biased by selected few samples. Lastly, for the 18 patients who were vaccinated after infection between TP3 and TP4, we evaluated the complete library of Spike-specific peptides only at TP4 and not at the earlier time points, thus, limiting the longitudinal analysis of some of the vaccine-induced T-cells across all time points in comparison to infection-induced T-cells.

In summary, by longitudinally profiling a large repertoire of SARS-CoV-2 epitopes, this study provides an integrated analysis of the frequency, specificity, and functional states of antigen-specific CD8^+^ T-cells during infection and memory formation. Our findings demonstrate that epitope-resolved analysis reveals quantitative and qualitative features of CD8^+^ T-cell immunity that may not be captured by bulk measurements. Thus, epitope-specific resolution of T-cell immunity may have a significant impact for therapeutic purposes, such as vaccine design, as well as in guiding future strategies involving novel pathogens.

## Contributors

A.O.G., S.R.H., and S.K.S. conceived the idea. S.P.A.H., D.S.H., S.R.H., and S.K.S. designed the study. S.P.A.H., M.K., and T.T. performed experiments. S.P.A.H., K.K.M., S.M.S.-G., K.D., A.G.P., and S.K.S performed data analysis and interpretation. S.P.A.H., K.K.M., S.M.S.-G., K.D., and A.G.P. prepared figures. D.S.H. facilitated patient recruitment, sample collection, and clinical data acquisition. A.O.G. supervised the clinical study, including patient participation, clinical data, and sample collection. S.P.A.H., K.D., A.G.P., and S.K.S. wrote the manuscript. S.K.S., and S.R.H supervised the study and verified the underlying data of this manuscript. All authors read, reviewed, and approved the final manuscript.

## Data sharing statement

The code for the mixed-effects logistic regression analyses is available at https://github.com/SKSgroup/COVID19_manuscript. The code for single-cell analysis is available at https://github.com/SKSgroup/sc-covid-cd8-profiling. Single-cell sequencing data have been deposited at https://doi.org/10.5281/zenodo.18759243. All other data are available in the main text or Supplementary Materials.

## Declaration of interests

Otto Mønsteds Fond Travel grant was paid to S.P.A.H., and subsequently transferred to the author's institution, in accordance with institutional policy. All other authors declare no competing interest.
