# Supplementary material for: Comprehensive longitudinal profiling identifies differential frequency, epitope specificity, and effector function of CD8+ T-cells across COVID-19 disease severities
Source: eBioMedicine. 2026 May 26;128:106310. doi: 10.1016/j.ebiom.2026.106310 (PMC13234737; doi:10.1016/j.ebiom.2026.106310)
Supplement: Supplementary Figures [file mmc2.docx]

**Supplementary Figures for**

**Comprehensive longitudinal profiling identifies differential frequency, epitope specificity, and effector function of CD8⁺ T-cells across COVID-19 disease severities**

Susana Patricia Amaya Hernandez^1^, Kamilla Kjærgaard Munk^1^, Konstantin Danilov^1^, Mohammad Kadivar^1^, Ditte Stampe Hersby^2^, Tripti Tamhane^1^, Simone Majken Stegenborg-Grathwohl^1^, Anders Gorm Pedersen^3^, Anne Ortved Gang^2,4^, Sine Reker Hadrup^1#^, Sunil Kumar Saini^1#*^

^1^Department of Health Technology, Section of Experimental and Translational Immunology, Technical University of Denmark, Kongens Lyngby, 2800, Denmark

^2^Department of Hematology, Copenhagen University Hospital, Rigshospitalet, Copenhagen, 2100, Denmark

^3^Department of Health Technology, Section for Bioinformatics, Technical University of Denmark, Kongens Lyngby, 2800, Denmark

^4^Department of Clinical Medicine, University of Copenhagen, Copenhagen, 2200, Denmark

^#^Equal contributions

*Corresponding author: Sunil Kumar Saini.

Email address: sukusa@dtu.dk

**This file includes:**

Figures S1 to S16

| **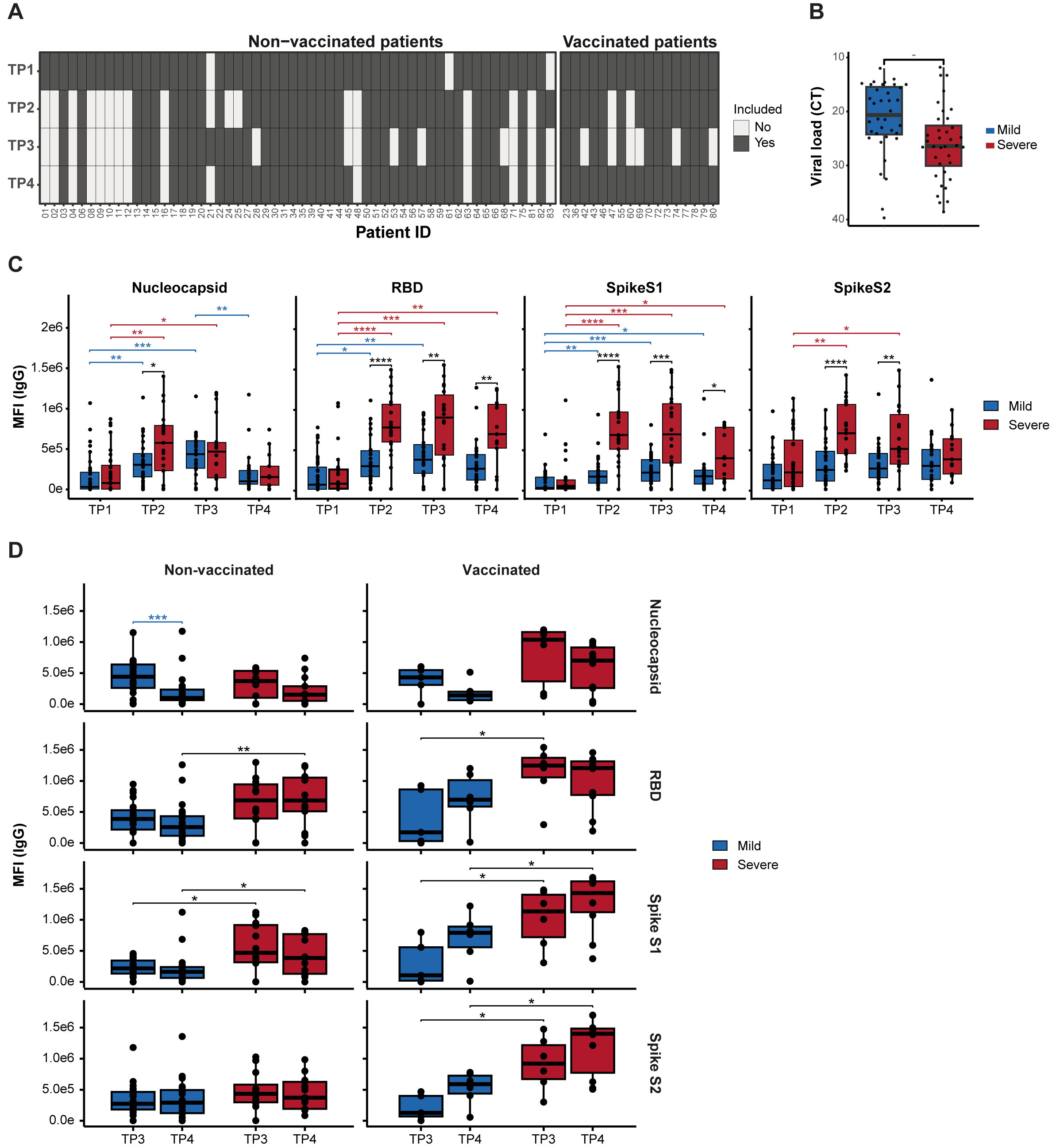** |
| --- |
| **Fig. S1. Clinical cohort overview and SARS-CoV-2 antibody responses in patients with COVID-19.** (**A**) PBMC sample availability across time points for individual patients with COVID-19. (**B**) SARS-CoV-2 viral load estimated from RT-PCR cycle threshold (Ct) values. Mann-Whitney test between disease severity, * (p ≤ 0.05). (**C**) Levels of IgG antibodies against the SARS-CoV-2 Spike protein subunits S1 and S2, the receptor-binding domain (RBD), and the nucleocapsid (N) protein in mild and severe patients with COVID-19 across time points TP1–TP4. TP4 samples from vaccinated individuals were excluded in this analysis to avoid confounding effects of vaccine-induced antibody responses on infection-induced levels. (**D**) Comparison between the levels of IgG antibodies against SARS-CoV-2 antigens for non-vaccinated and vaccinated patients with mild and severe COVID-19 between TP3 and TP4. (**C**, **D**) Mann-Whitney test between disease severity and Mann-Whitney test adjusting p-values with the Bonferroni method for comparison between time points, **** (p < 0.0001), *** (p < 0.001), ** (p < 0.01) and * (p ≤ 0.05). |

| 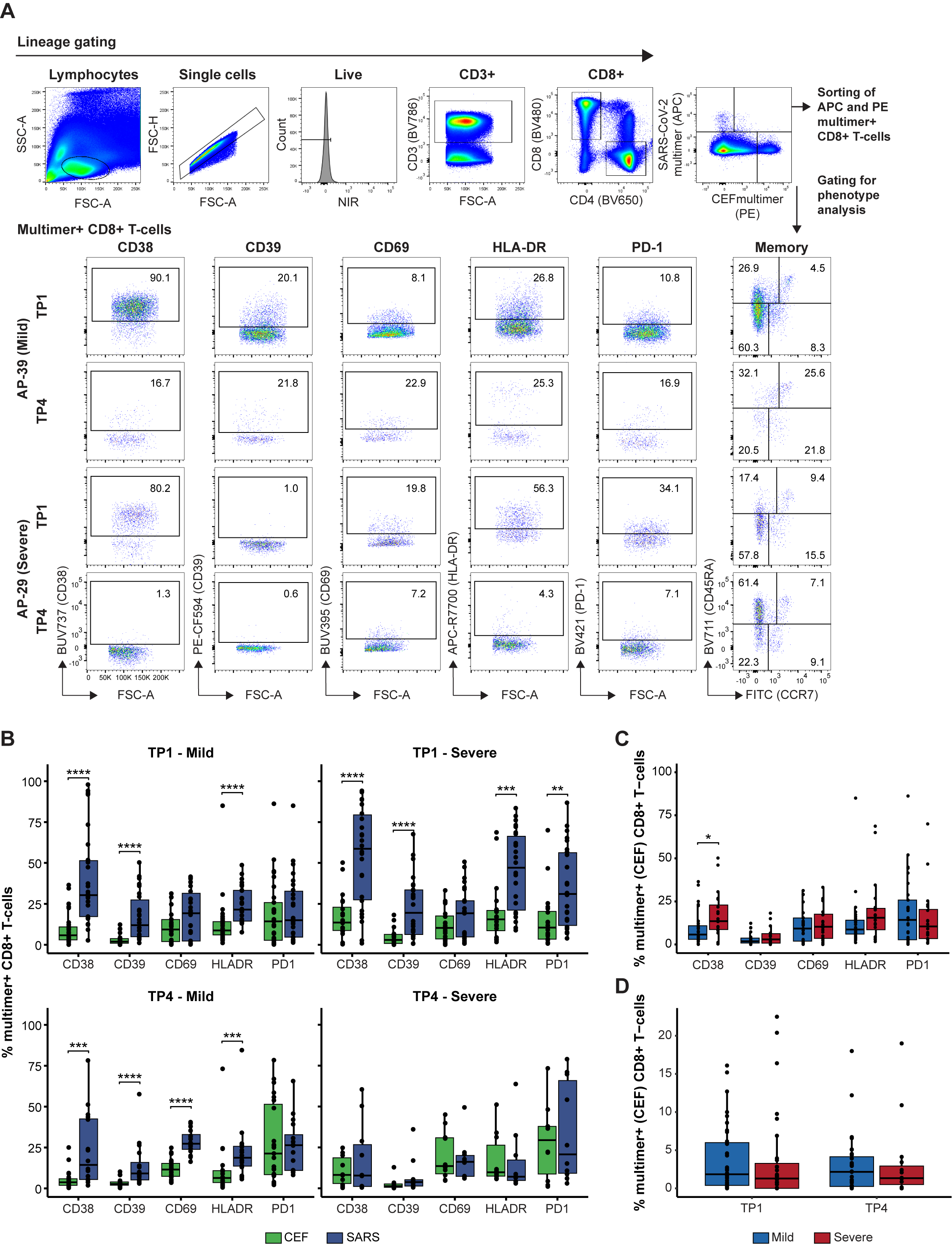 |  |  |
| --- | --- | --- |
| **Fig. S2. Flow cytometry-based identification and comparison of SARS-CoV- and CEF-specific CD8^+^ T-cells.** (**A**) Representative flow cytometry plots illustrating the gating strategy applied to PBMCs from patients with COVID-19. PBMCs were stained with DNA-barcoded pHLA multimers and surface antibody markers to identify SARS-CoV-2 (APC)– and CEF (PE)–multimer⁺ CD8⁺ T-cells and to quantify the expression of phenotypic markers within the multimer⁺ CD8⁺ T-cell populations. (**B**) Box plot compares the percentage of SARS-CoV-2 and CEF pHLA multimer^+^ CD8^+^ T-cells expressing the indicated surface markers at TP1 and TP4. (**C**) Comparison of the percentage of CEF pHLA multimer^+^ CD8^+^ T-cells expressing the indicated surface markers between the mild and severe COVID-19 groups at TP1. (**D**) Box plot compares the frequency (% of multimer^+^ CD8^+^ T-cells) of CEF-specific T-cell populations between patients with mild and severe COVID-19 at TP1 and TP4. |  |  |
| 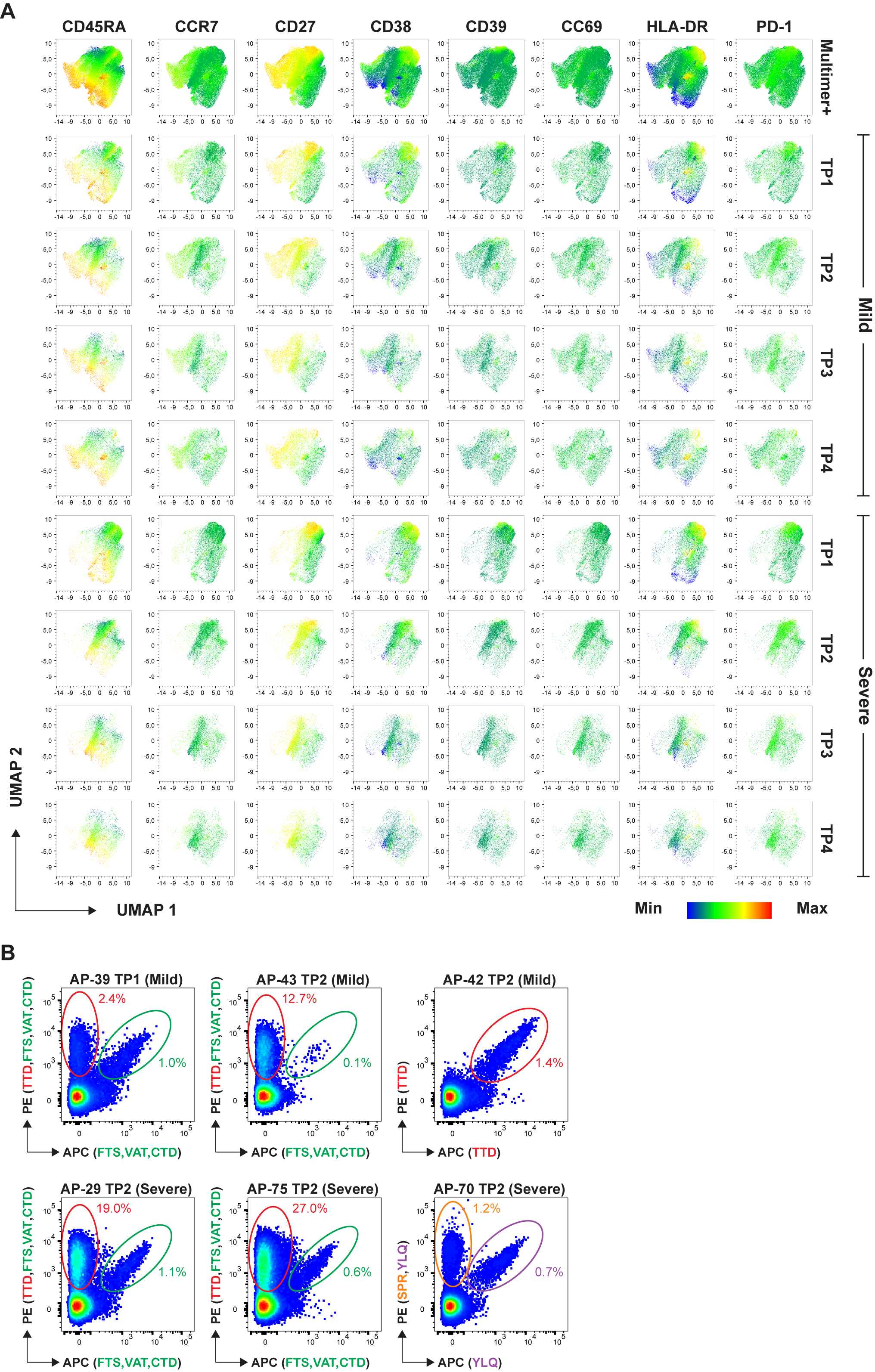 | | |
| **Fig. S3. Phenotypic characterisation and pHLA multimer–based identification of SARS-CoV-2–specific CD8⁺ T-cell populations.** (**A**) UMAP showing the expression of individual phenotype markers CD45-RA, CCR7, CD27, CD38, CD39, CD69, HLA-DR, and PD-1 for SARS-CoV-2 pHLA multimer^+^ CD8^+^ T-cells in COVID-19 patients for all multimer^+^ T-cells (top row) and separated by severity and time point. (**B**) Validation of estimated frequencies of SARS-CoV-2 epitope-specific CD8^+^ T-cells identified by DNA-barcoded pHLA multimer analysis, using conventional fluorophore-labelled pHLA multimers. Flow cytometry plots show PBMCs stained with single- or dual-fluorophore labelled pHLA multimers for selected immunodominant SARS-CoV-2 epitopes, with frequencies reported as % of total CD8^+^ T-cells. Gated populations (corresponding to the epitope-specificity labelled on the X- and Y-axis) represent T-cells stained with a single-fluorochrome, whereas diagonal double-positive populations reflect dual-colour staining. pHLA multimers for FTS, VAT, and CTD were tested as a combined pool and labelled with the same fluorophore combination (PE and APC), hence reflects combined frequency of T-cells reactive to these three epitopes*.* Abbreviations: TTD, TTDPSFLGRY; FTS, FTSDYYQLY; VAT, VATSRTLSYY; CTD, CTDDNALAYY; YLQ, YLQPRTFLL; SPR, SPRWYFYYL. | | |
| **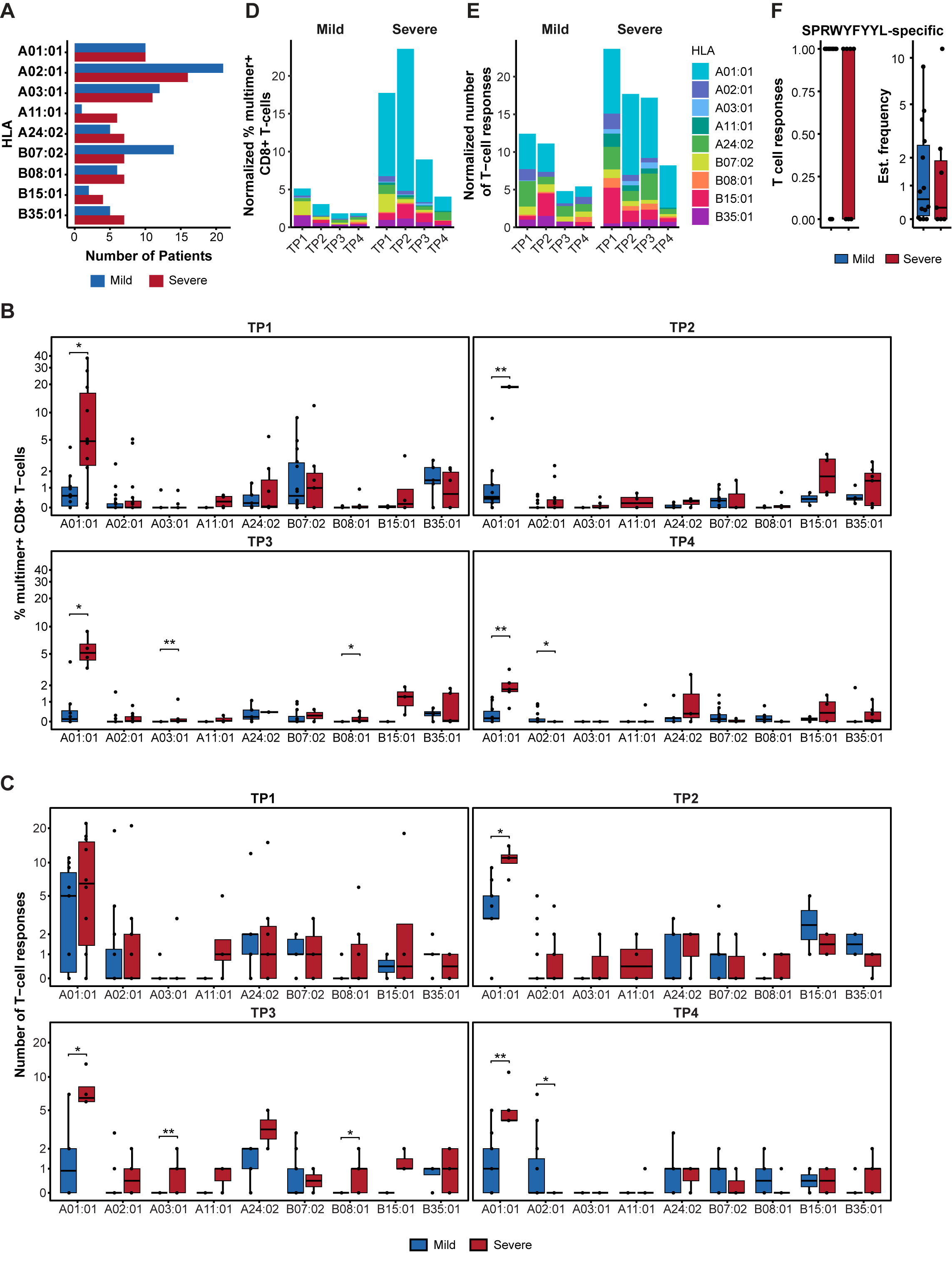** | |  |
| **Fig. S4. HLA-specific distribution of patient cohort and the identified SARS-CoV-2-specific T-cells in mild and severe COVID-19.** (**A**) Number of patients with severe and mild disease included for each HLA allele analysed in this study. (**B**, **C**) Box plots comparing the sum of estimated frequencies (%) (**B**) and the total number (**C**) of SARS-CoV-2-specific T-cell populations restricted to each HLA allele between COVID-19 severity groups across four time points post-diagnosis. (**D**, **E**) Stacked bar plots summarize the normalised estimated frequency (**D**) and the normalised number (**E**) of SARS-CoV-2-specific T-cell populations per HLA allele, across four time points (TP1–TP4) in patients with mild or severe disease. Normalisation was performed based on the number of patients carrying each HLA allele at each time point within each disease severity group. (**F**) Comparison of HLA-B07:02-restricted SPRWYFYYL-specific T-cell populations, shown as total responses and estimated frequencies (%), between patients with mild and severe disease at TP1. (B, C, F) Mann-Whitney test, **** (p < 0.0001), *** (p < 0.001), ** (p < 0.01) and * (p ≤ 0.05). | | |
| 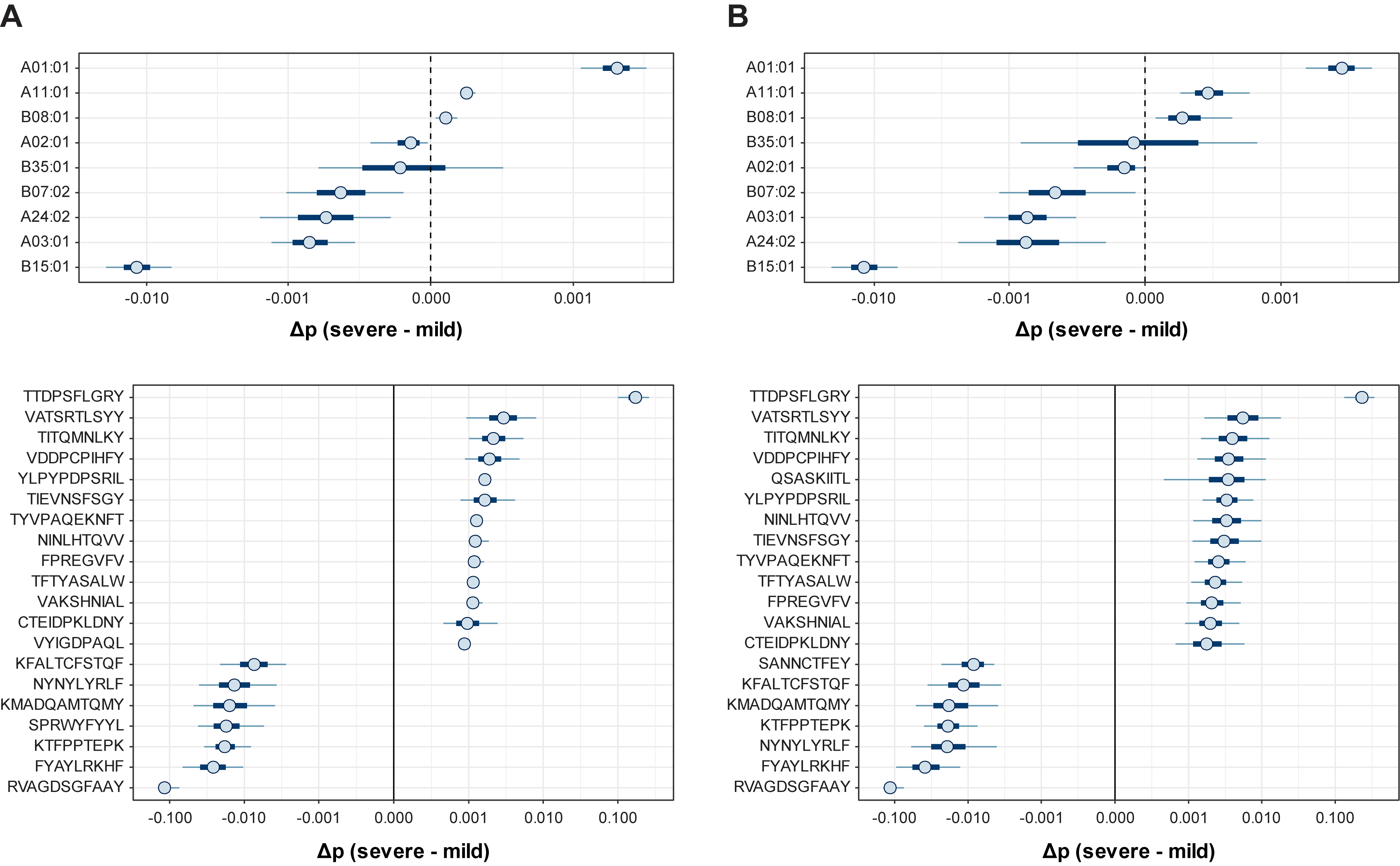 |  |  |
| **Fig. S5.** **Sensitivity analyses of the mixed-effects regression model showing HLA allele–specific and peptide–HLA pair–specific and APC values.** (**A**) Model refitted after exclusion of two asymptomatic patients with mild disease. (**B**) Model refitted with adjustment for three broad comorbidity classes. |  |  |

| \| **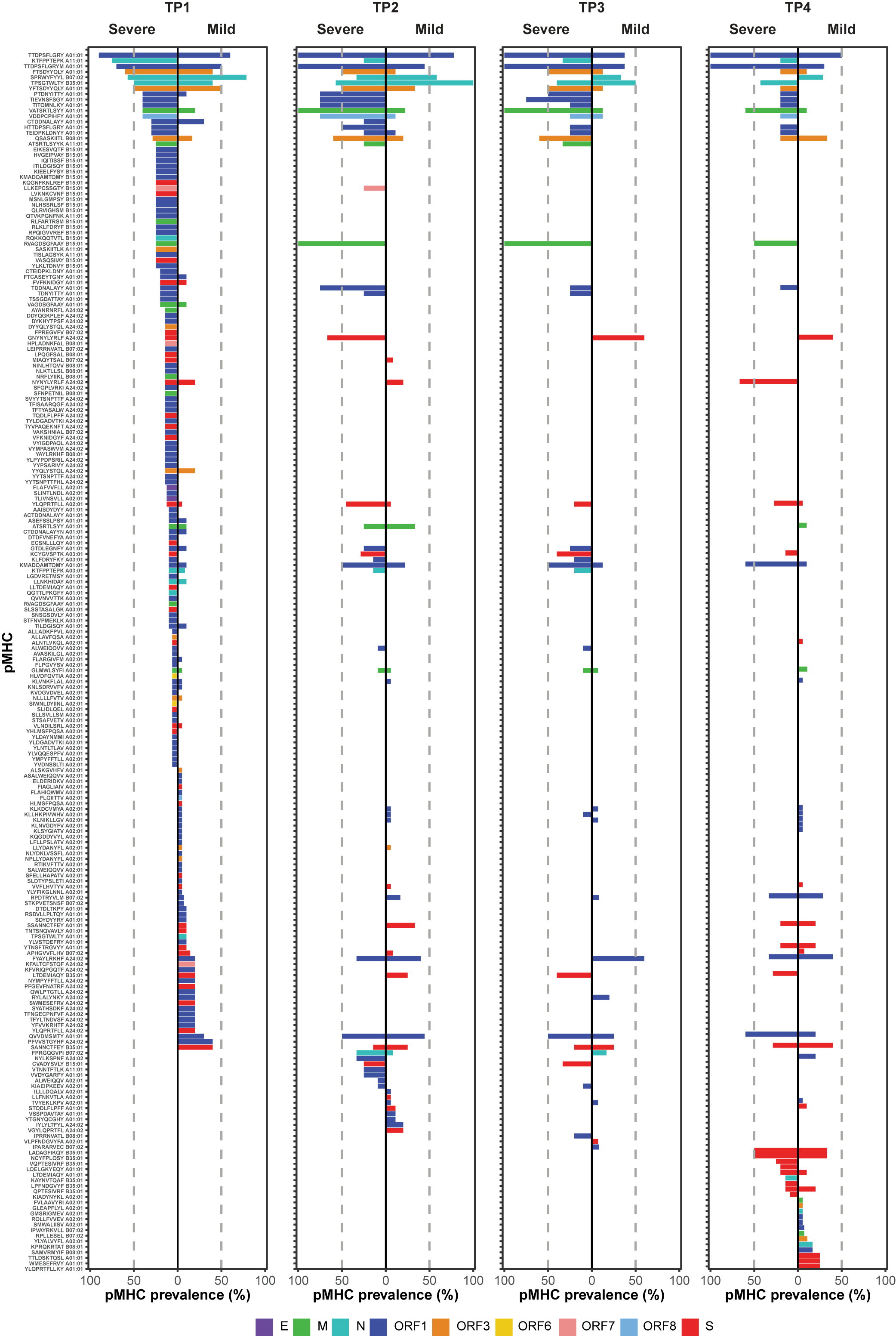** \| \| --- \| \| **Fig. S6. Prevalence of CD8^+^ T-cell recognition towards SARS-CoV-2 epitopes.** Prevalence of T-cell recognition of individual epitopes detected in patients with COVID-19 split according to disease severity. TP4 prevalence includes T-cell recognition towards additional Spike-derived peptides tested only in vaccinated patients. Only pHLA tested in more than 2 donors were included in this analysis. A dotted line is placed at 50% of prevalence to distinguish immunodominant epitopes. Bars are coloured according to their protein of origin. \| |
| --- | --- | --- |
| **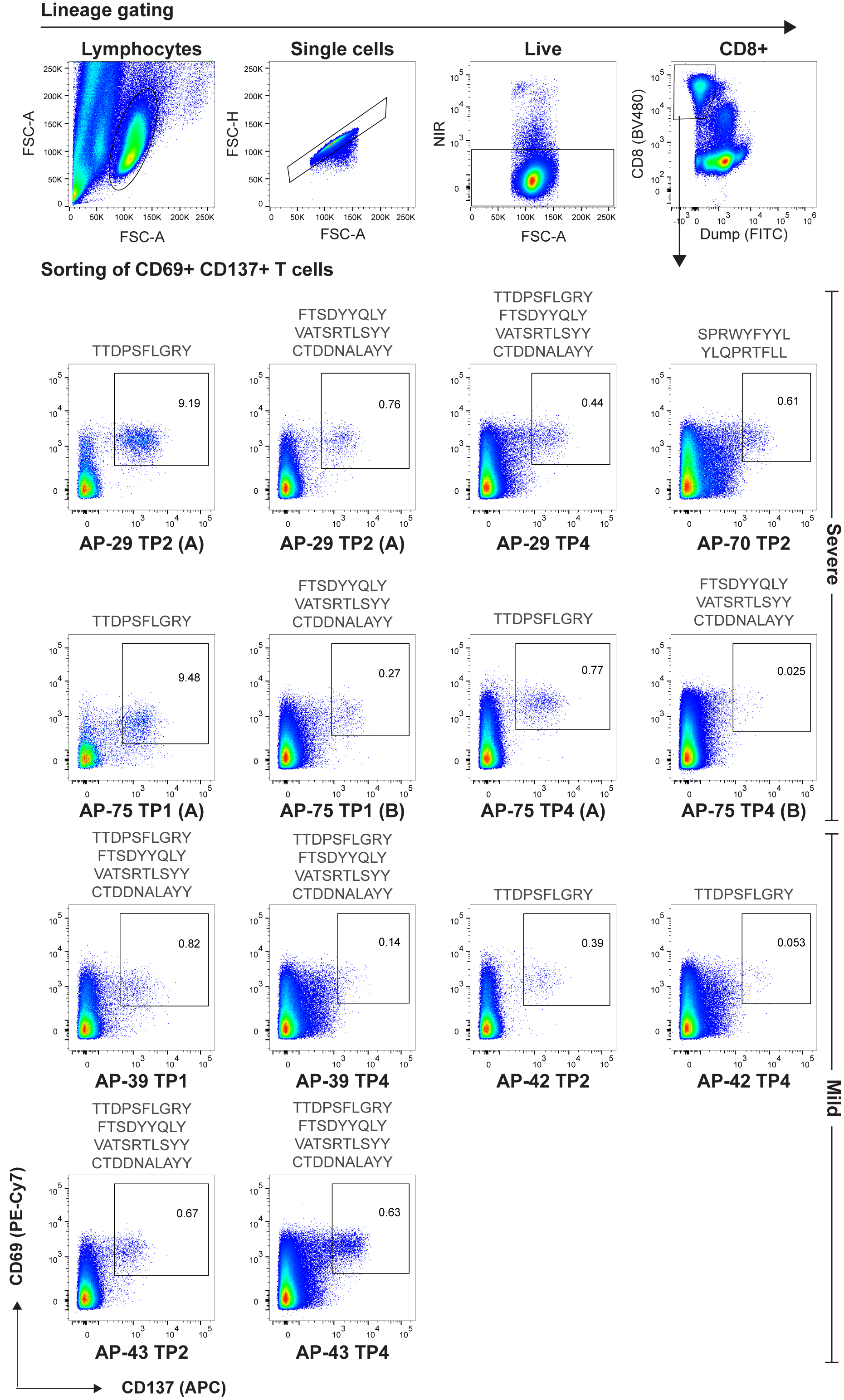** |
| **Fig. S7. Gating strategy for sorting activated (CD69^+^ CD137^+^) CD8^+^ T-cells for single-cell analysis.** Representative flow cytometry plots showing the gating strategy on PBMCs from patients with COVID-19 to sort double positive CD69^+^ CD137^+^ T-cells used for single-cell analysis. Dot plots show the percentage of CD69^+^ CD137^+^ T-cell populations sorted from each sample. |
| **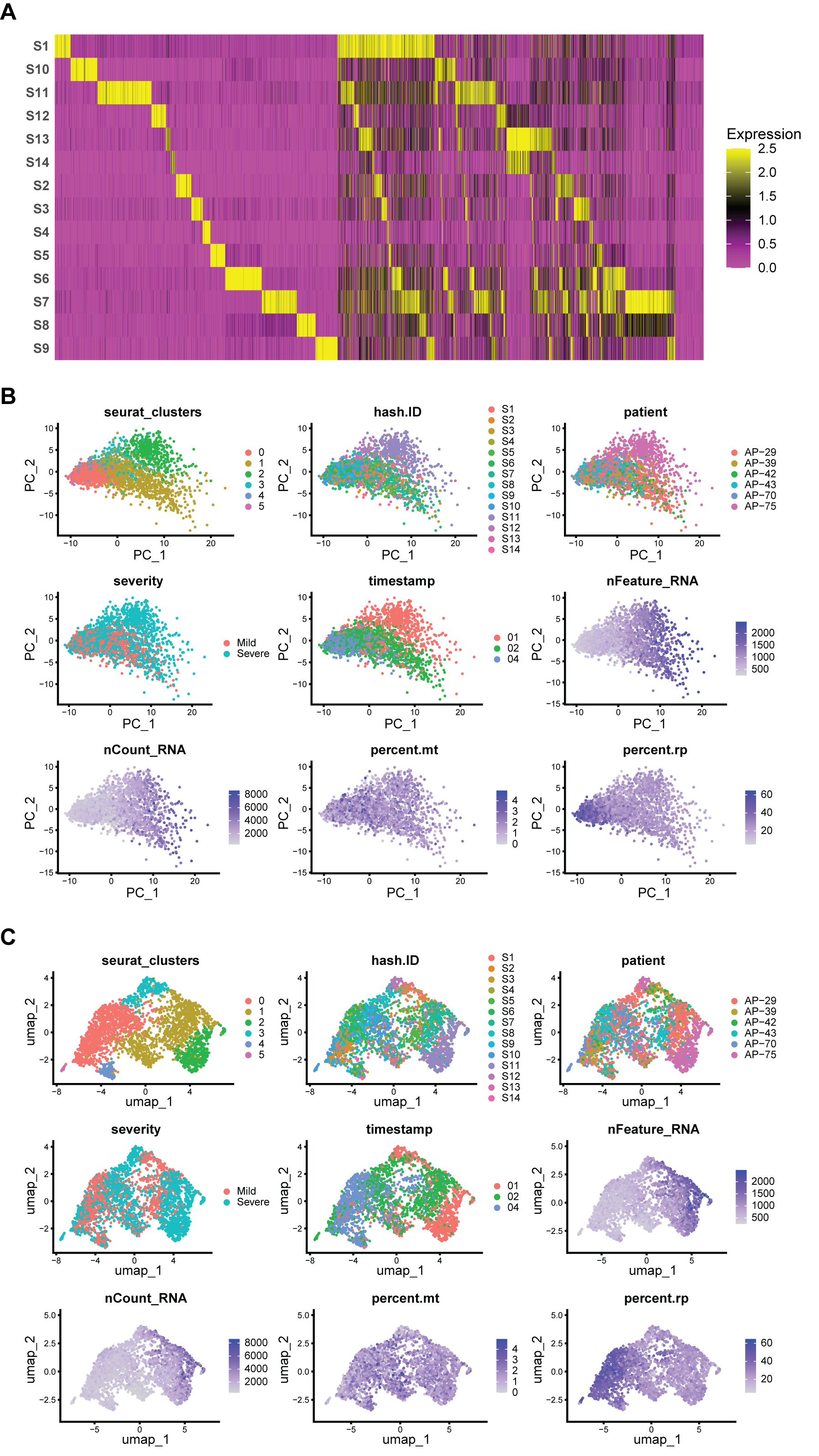** |
| **Fig. S8.** **Demultiplexing and single-cell transcriptome analysis.** (**A**) HTO heatmap after demultiplexing for all samples. (**B**) PCA plots for a selected set of variables. (**C**) UMAP plots for the selected set of variables. PCA: principal component analysis, HTO: hashtag oligo, UMAP: uniform manifold approximation and projection. |
| **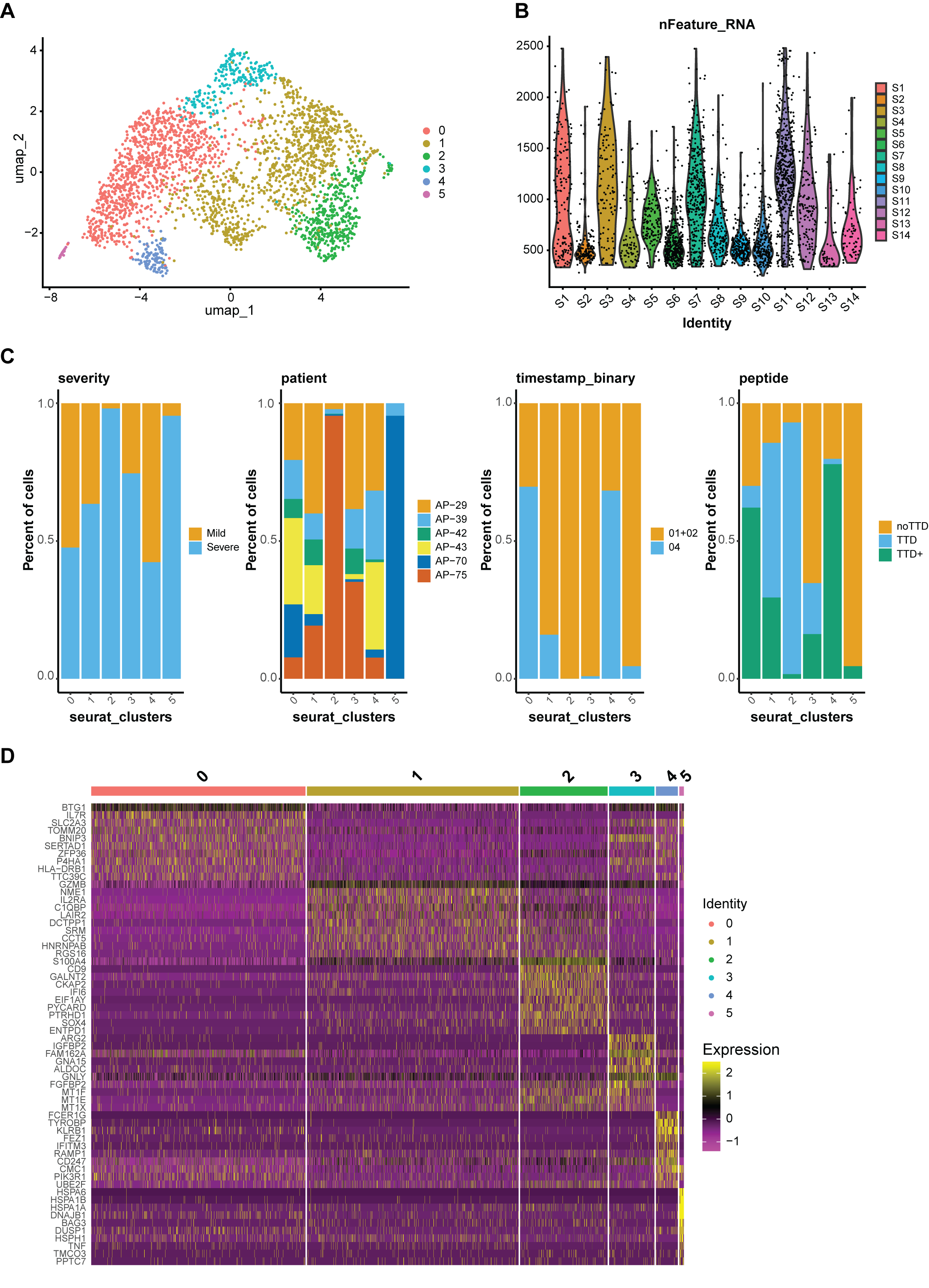** |
| **Fig. S9. Clinical and transcriptomic characteristics of single-cell clusters.** (**A**) UMAP of resulting clusters generated for all samples. (**B**) Distribution of nFeature_RNA across samples. (**C**) Distribution of clinical and experimental variables across clusters. (**D**) Heatmap of top 10 markers for each cluster. |

| **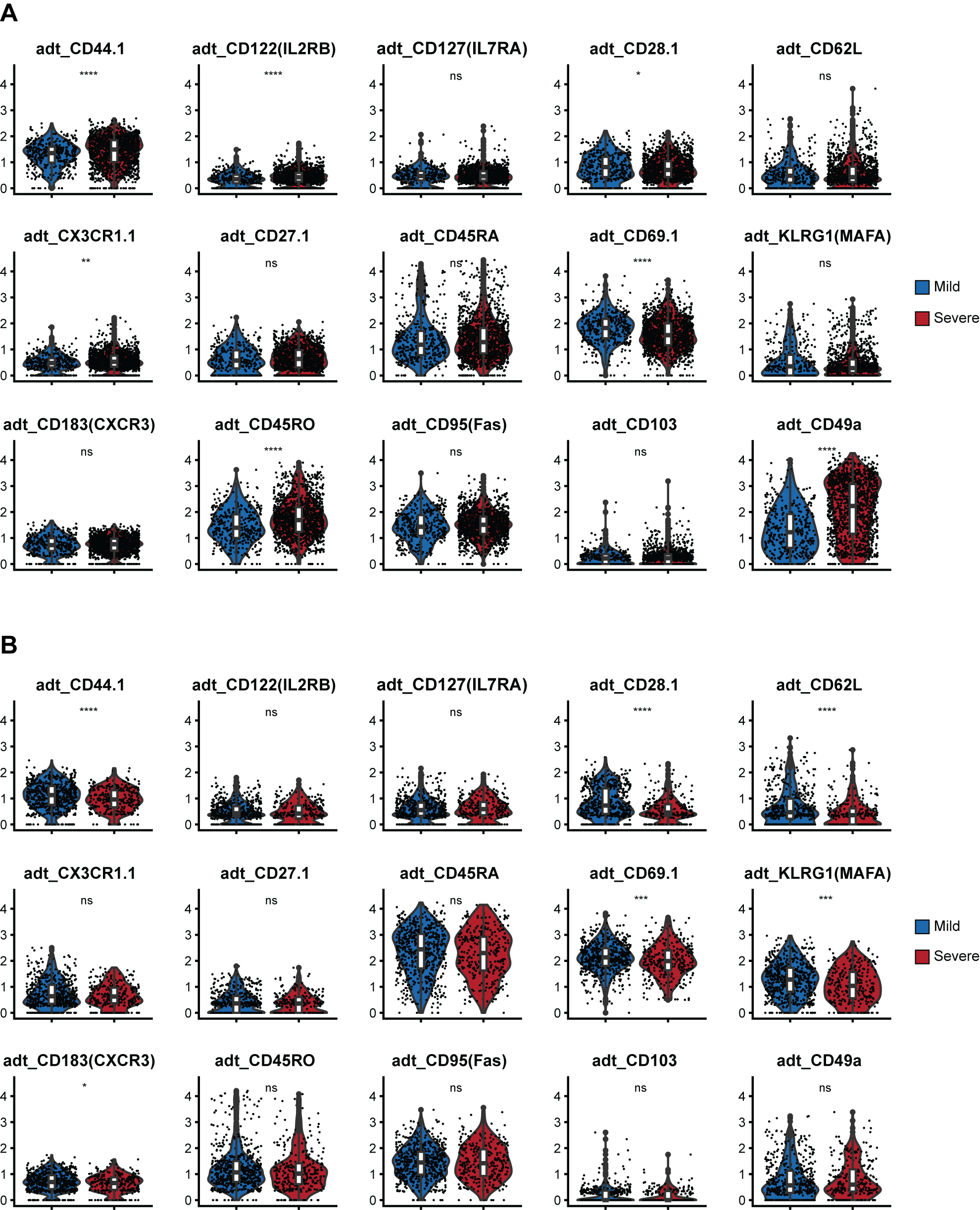** |
| --- |
| **Fig. S10. Comparison of markers’ expression levels.** (**A**, **B**) Violin plots comparing the expression levels of selected memory markers (surface markers) between mild and severe samples in early time point (**A**) and in late time point (**B**). Mann-Whitney test: *p < 0.05, **p < 0.01, ***p < 0.001, ****p < 0.0001. |

| **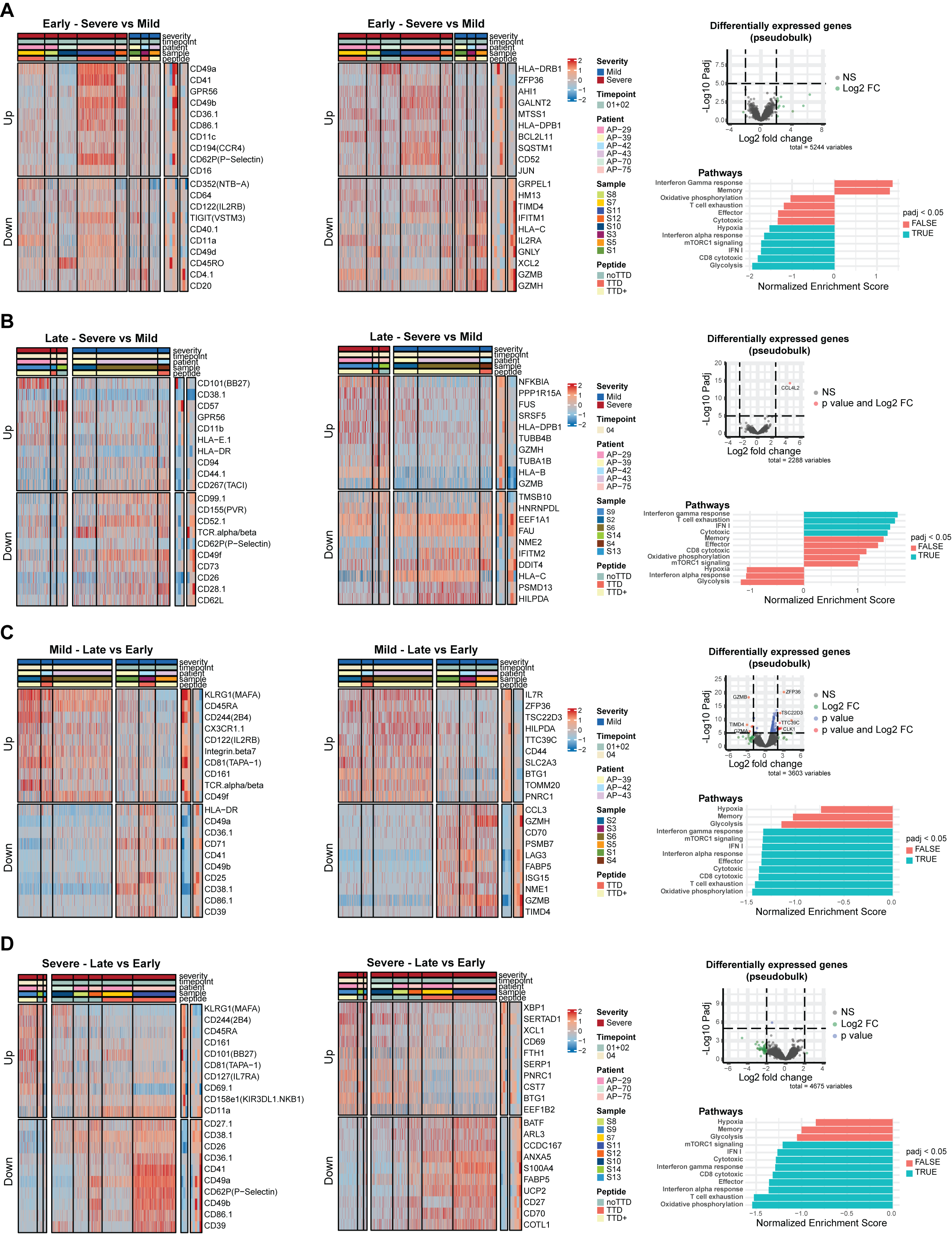** |
| --- |
| **Fig. S11. Differential expression analysis of samples between different conditions.** (**A-D**) Differential expression analysis on a single-cell level for surface markers (**left heatmap**) and gene expressions (**right heatmap**). Top 10 markers and genes were selected from each side (avg_log2FC). Volcano plots (**right, top**) represent the results of pseudobulk differential expression gene analysis. Gene set enrichment analysis for selected gene sets (**right, bottom**). (**A**, **B**) Comparison of patients with severe vs patients with mild disease in early (**A**) and late (**B**) COVID-19. (**C**, **D**) Comparison of late vs early COVID-19 in patients with mild (**C**) and severe (**D**) disease. TTD: TTDPSFLGRY; TTD+: TTDPSFLGRY + other peptides; noTTD: any peptide other than TTDPSFLGRY. |
| **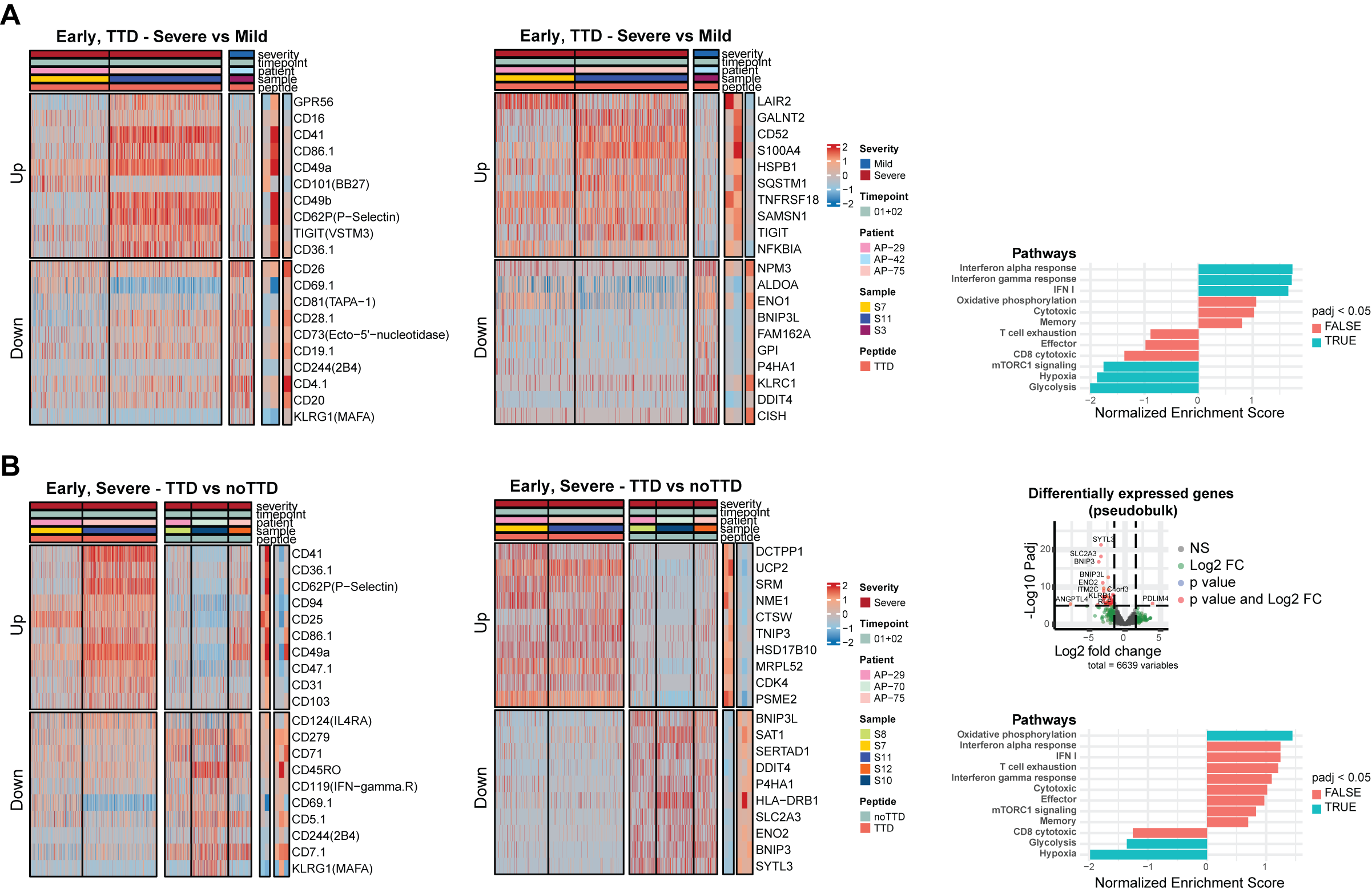** |
| **Fig. S12. Differential expression analysis in early (TP1/TP2) samples between different conditions.** (**A-B**) Differential expression analysis on a single-cell level for surface markers (**left heatmap**) and gene expressions (**right heatmap**). Top 10 markers and genes were selected from each side (avg_log2FC). Volcano plots represent results of pseudobulk differential expression gene analysis (**right, top**). Gene set enrichment analysis for selected gene sets (**right, bottom**). (**A**) Comparison of patients with severe vs mild disease in early COVID-19, T-cells stimulated with TTD peptide. (**B**) Comparison of T-cells stimulated with TTD vs other peptides, in patients with severe disease at late COVID-19. TTD: TTDPSFLGRY; noTTD: any peptide other than TTDPSFLGRY. |

| **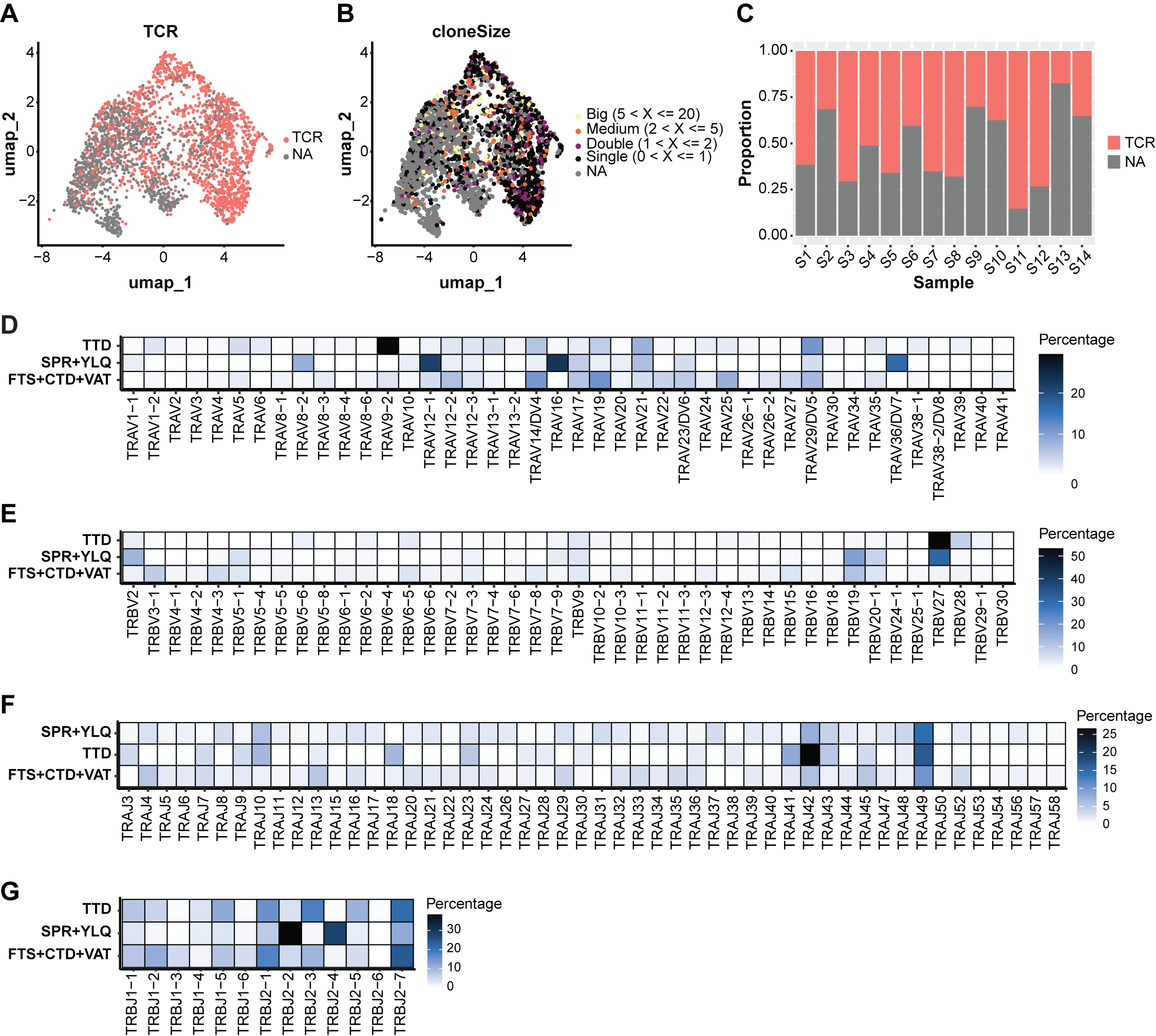** |
| --- |
| **Fig. S13. Single-cell TCR sequencing analysis of SARS-CoV-2-specific responses. (A)** UMAP representation of all samples, coloured by the availability of TCR information. (**B**) UMAP representation of all samples, colored by clone size. (**C**) Bar plot showing the distribution of TCR information availability across all samples. Heatmaps of the relative usage of V genes in alpha (**D**) and beta (**E**) chains for samples stimulated with various SARS-CoV-2 peptides. Heatmaps of the relative usage of J genes in alpha (**F**) and beta (**G**) chains for early time point samples stimulated with various SARS-CoV-2 peptides. TTD: TTDPSFLGRY, FTS: FTSDYYQLY, VAT: VATSRTLSYY, CTD: CTDDNALAYY, YLQ: YLQPRTFLL, SPR: SPRWYFYYL. |

| **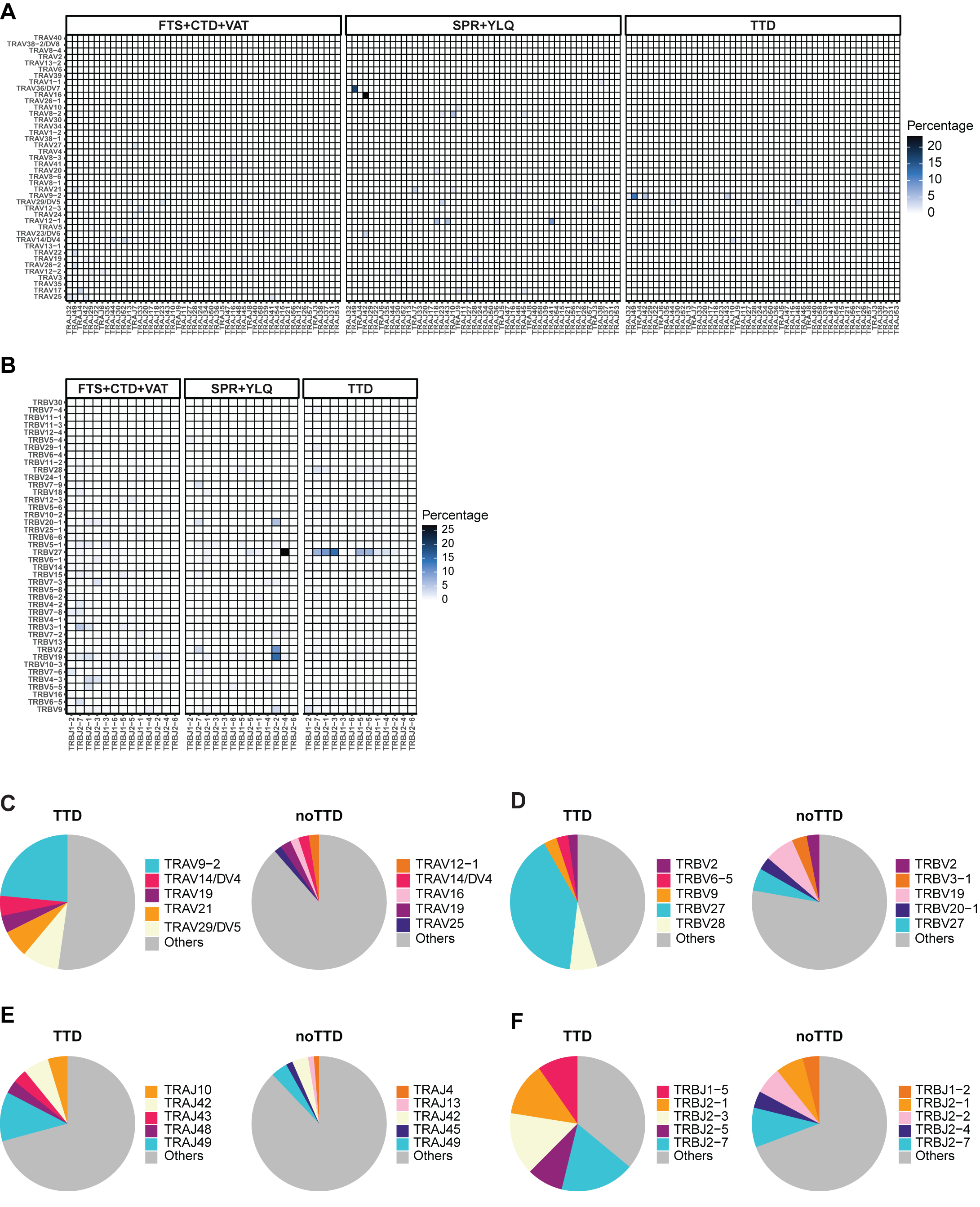** |
| --- |
| **Fig. S14. Analysis of V and J gene usage in TCR alpha and beta chains for SARS-CoV-2-specific populations in early time points**. (**A**, **B**) Heatmaps showing the relative usage of V and J gene combinations in the TCR alpha (**A**) and beta (**B**) chains for early-phase samples stimulated with SARS-CoV-2 peptides. (**C**, **D**) Pie charts summarizing the top 5 most frequently used V genes in TTD versus no TTD samples for alpha (**C**) and beta (**D**) chains. (**E**, **F**) Pie charts summarizing the top 5 J most frequently genes used in TTD versus no TTD samples at early time points for alpha (**E**) and beta (**F**) chains. TTD: TTDPSFLGRY, FTS: FTSDYYQLY, VAT: VATSRTLSYY, CTD: CTDDNALAYY, YLQ: YLQPRTFLL, SPR: SPRWYFYYL. |

| **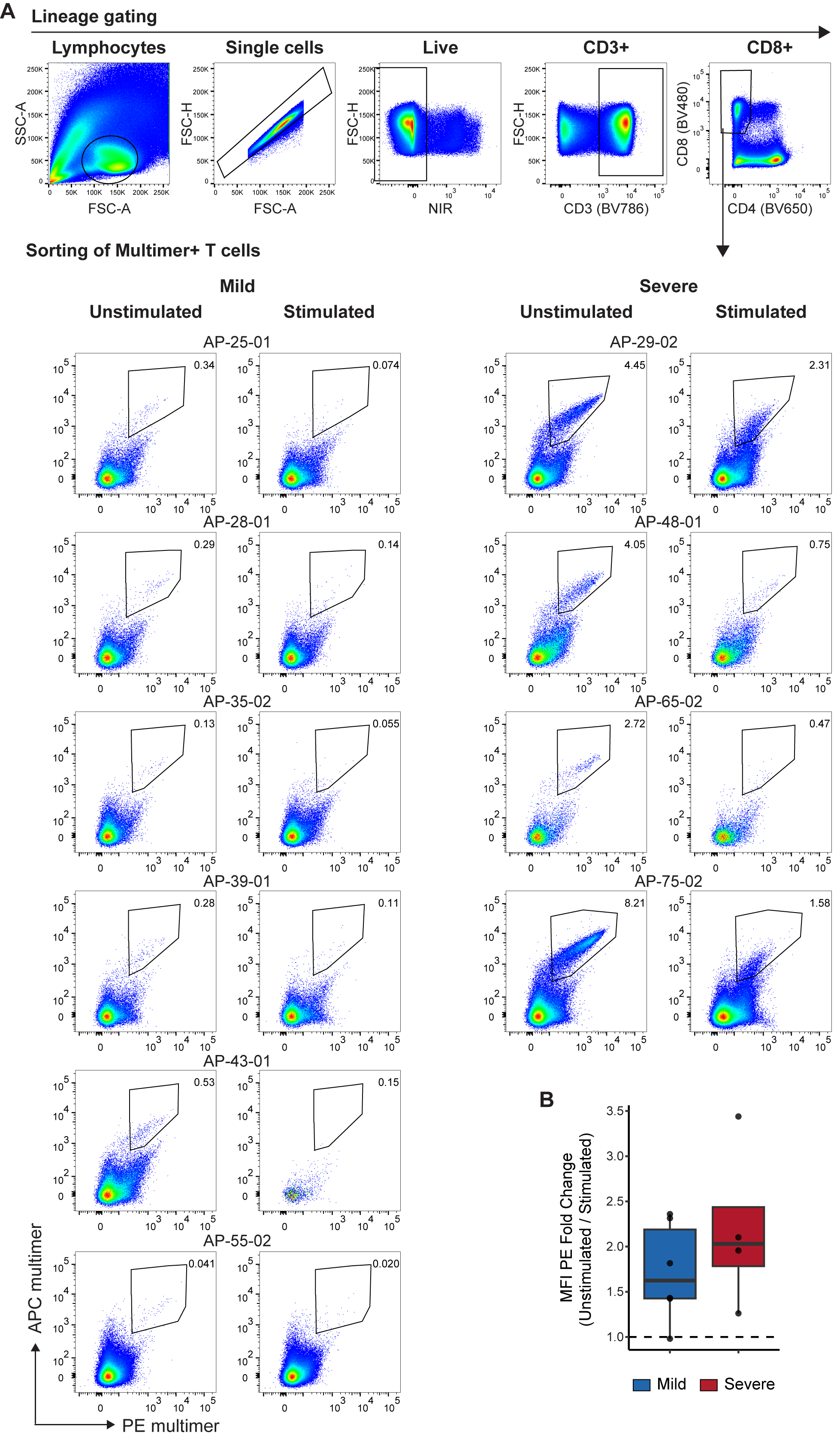** |
| --- |
| **Fig. S15. Analysis of TCR down-regulation in multimer⁺ CD8⁺ T-cell populations following peptide stimulation. (A)** Representative flow cytometry plots demonstrating the gating strategy applied to PBMCs from patients with COVID-19, either unstimulated or peptide-stimulated for the identification and sorting of double-positive (PE^+^ APC^+^) multimer^+^ CD8^+^ T-cell populations. Dot plots display the percentages of PE^+^ APC^+^ multimer^+^ T-cells sorted from each patient sample, stratified by disease severity. Sorted cells were used for subsequent analysis of TCR down-regulation following peptide stimulation. (**B**) Box plot showing the fold change in MFI (mean fluorescence intensity) of multimer⁺ CD8⁺ T-cell populations following peptide stimulation, calculated as the ratio of unstimulated to stimulated samples. Values above or below the dashed line indicate decreased or increased PE MFI following stimulation, respectively. No statistically significant difference was observed between disease severity groups (Mann–Whitney test). |
| \| 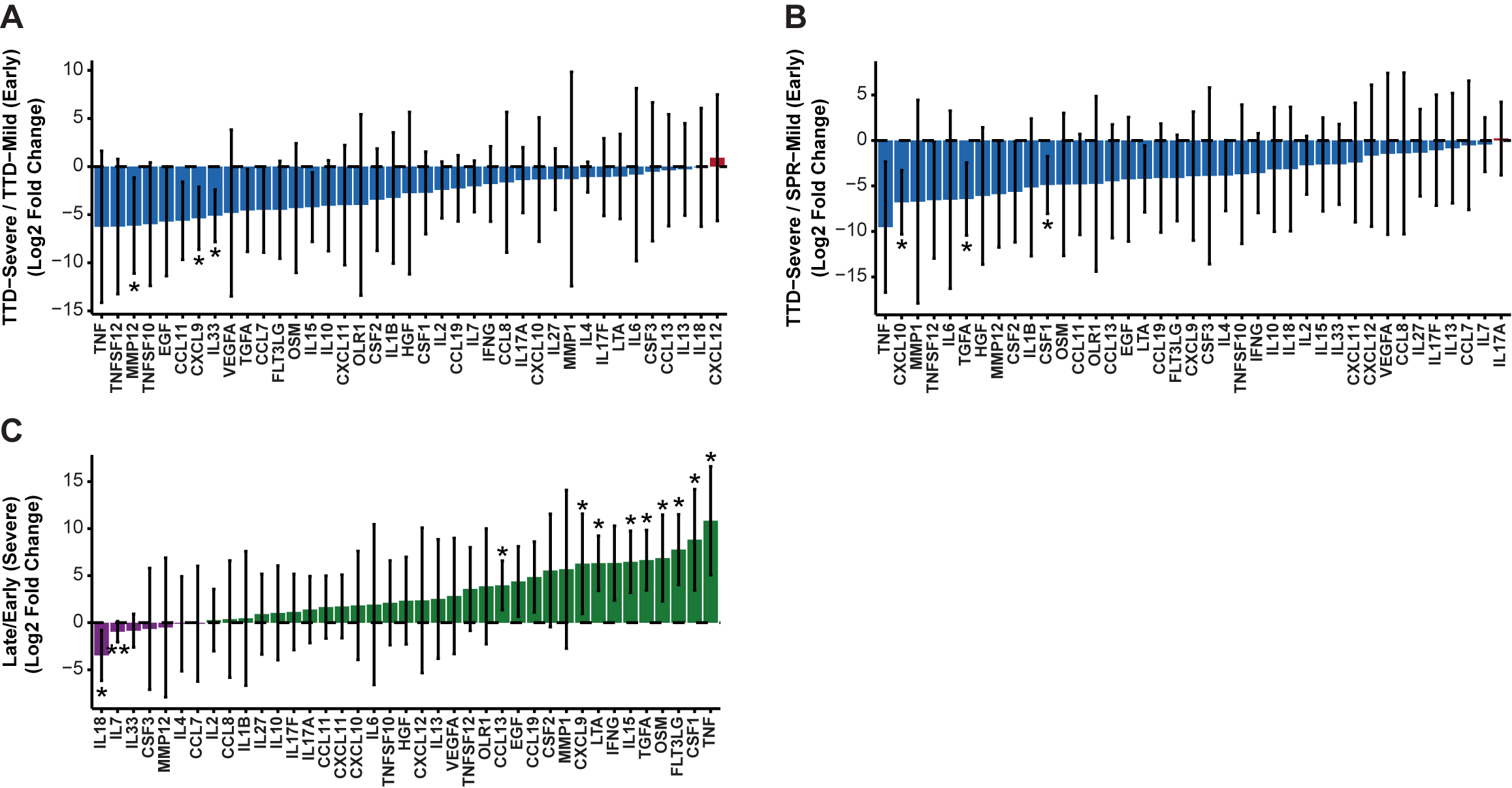 \| \| --- \| \| **Fig. S16. Comparison of cytokines and chemokines secretion by epitope-specific T-cells after peptide stimulation using Olink assay.** Bar plots show log2 fold changes in mean protein secretion levels, with error bars indicating 95% confidence intervals. (**A**) Severe-to-mild protein secretion ratio in PBMCs stimulated with the TTD peptide (TTDPSFLGRY) using early-phase COVID-19 patient samples. (**B**) Protein secretion ratios comparing PBMCs from patients with severe disease stimulated with the TTD peptide (TTDPSFLGRY) and PBMCs from patients with mild disease stimulated with the SPR peptide (SPRWYFYYL), using early-phase samples. (C) Late-to-early protein secretion ratio in PBMCs from patients with severe disease following peptide stimulation. Statistical significance was assessed using the Mann–Whitney test; ** p < 0.01, * p ≤ 0.05. \| |
